# An Invertebrate Warburg Effect: A Shrimp Virus Achieves Successful Replication by Altering the Host Metabolome via the PI3K-Akt-mTOR Pathway

**DOI:** 10.1371/journal.ppat.1004196

**Published:** 2014-06-12

**Authors:** Mei-An Su, Yun-Tzu Huang, I-Tung Chen, Der-Yen Lee, Yun-Chieh Hsieh, Chun-Yuan Li, Tze Hann Ng, Suh-Yuen Liang, Shu-Yu Lin, Shiao-Wei Huang, Yi-An Chiang, Hon-Tsen Yu, Kay-Hooi Khoo, Geen-Dong Chang, Chu-Fang Lo, Han-Ching Wang

**Affiliations:** 1 Institute of Biotechnology, College of Bioscience and Biotechnology, National Cheng Kung University, Tainan, Taiwan; 2 Institute of Zoology, College of Life Science, National Taiwan University, Taipei, Taiwan; 3 Institute of Biochemical Sciences, College of Life Science, National Taiwan University, Taipei, Taiwan; 4 Center for Systems Biology, National Taiwan University, Taipei, Taiwan; 5 Core Facilities for Protein Structural Analysis, Institute of Biological Chemistry, Academia Sinica, Taipei, Taiwan; 6 Academia Sinica Common Mass Spectrometry Facilities at Institute of Biological Chemistry, Taipei, Taiwan; 7 Department of Life Science, College of Life Science, National Taiwan University, Taipei, Taiwan; 8 Institute of Bioinformatics and Biosignal Transduction, National Cheng Kung University, Tainan, Taiwan; University of Washington, United States of America

## Abstract

In this study, we used a systems biology approach to investigate changes in the proteome and metabolome of shrimp hemocytes infected by the invertebrate virus WSSV (white spot syndrome virus) at the viral genome replication stage (12 hpi) and the late stage (24 hpi). At 12 hpi, but not at 24 hpi, there was significant up-regulation of the markers of several metabolic pathways associated with the vertebrate Warburg effect (or aerobic glycolysis), including glycolysis, the pentose phosphate pathway, nucleotide biosynthesis, glutaminolysis and amino acid biosynthesis. We show that the PI3K-Akt-mTOR pathway was of central importance in triggering this WSSV-induced Warburg effect. Although dsRNA silencing of the mTORC1 activator Rheb had only a relatively minor impact on WSSV replication, *in vivo* chemical inhibition of Akt, mTORC1 and mTORC2 suppressed the WSSV-induced Warburg effect and reduced both WSSV gene expression and viral genome replication. When the Warburg effect was suppressed by pretreatment with the mTOR inhibitor Torin 1, even the subsequent up-regulation of the TCA cycle was insufficient to satisfy the virus's requirements for energy and macromolecular precursors. The WSSV-induced Warburg effect therefore appears to be essential for successful viral replication.

## Author Summary

The Warburg effect (or aerobic glycolysis) is a metabolic shift that was first found in cancer cells, but has also recently been discovered in vertebrate cells infected by viruses. The Warburg effect facilitates the production of more energy and building blocks to meet the enormous biosynthetic requirements of cancerous and virus-infected cells. To date, all of our knowledge of the Warburg effect comes from vertebrate cell systems and our previous paper was the first to suggest that the Warburg effect may also occur in invertebrates. Here, we use a state-of-the-art systems biology approach to show the global metabolomic and proteomic changes that are triggered in shrimp hemocytes by a shrimp virus, white spot syndrome virus (WSSV). We characterize several critical metabolic properties of the invertebrate Warburg effect and show that they are similar to the vertebrate Warburg effect. WSSV triggers aerobic glycolysis via the PI3K-Akt-mTOR pathway, and during the WSSV genome replication stages, we show that the Warburg effect is essential for the virus, because even when the TCA cycle is boosted in mTOR-inactivated shrimp, this fails to provide enough energy and materials for successful viral replication. Our study provides new insights into the rerouting of the host metabolome that is triggered by an invertebrate virus.

## Introduction

The Warburg effect, which was first described by Warburg in the 1930s, is a metabolic rerouting used by tumor cells and cancer cells to support their high energy requirements and high rates of macromolecular synthesis [1], [2]. In cancer cells, the main hallmark of the Warburg effect is aerobic glycolysis, in which glucose consumption and lactate production are both increased even in the presence of oxygen [3]. Several other metabolic pathways are also enhanced, including the pentose phosphate pathway (PPP), amino acid metabolism and lipid homeostasis. The Warburg effect can also be induced *in vitro* by some vertebrate viruses, including human papillomavirus (HPV) [4]; human cytomegalovirus (HCMV) [5], [6], Kaposi's sarcoma herpesvirus (KSHV) [7] and hepatitis C virus (HCV) [8], and recently we reported an *in vivo* Warburg-like effect that was induced in shrimp hemocytes by the white spot syndrome virus (WSSV; genus *Whispovirus*, family *Nimaviridae*) [9].

WSSV is a large unique, complex, dsDNA virus, and in shrimp hemocytes, its complete *in vivo* replication cycle takes 22–24 h [9], [10]. Although over 90% of WSSV viral genes show no sequence homology to any other known genes, some of its genes are known to express at different times in its replication cycle, including the immediate early gene *ie1*, the early gene DNA polymerase (*dna pol*), the late structural protein gene *vp28* and the very late DNA mimic protein gene *icp11*. Our previous study showed that WSSV induced the hallmarks of metabolic changes associated with the mammalian Warburg effect at the beginning of its genome replication stage (12 hours post injection [hpi]) [9]. However, since this was the first time that an invertebrate virus had been shown to produce this kind of effect, in the present paper, we look more closely at the global metabolomic and proteomic changes induced by WSSV in order to confirm that all of the interrelated metabolic effects seen in vertebrate cells are also found in the invertebrate Warburg effect. For the metabolomic study, we used liquid chromatography-electrospray ionization-tandem mass spectrometry (LC-ESI-MS/MS) to identify and measure the levels of intracellular metabolites in shrimp hemocytes at 12 and 24 hpi, i.e. at the beginning and end of WSSV's genome replication cycle. For the proteomic profiling, we used label-free proteomics at the same time points. Since activation of the PI3K-Akt-mTOR signaling pathway is used by cancer cells and viruses to trigger the Warburg effect [11]–[13], and it is also required for the effective replication of vertebrate viruses [14]–[17], in the second part of this study, we use *in vivo* drug treatments to investigate whether WSSV also uses this signal pathway to trigger the Warburg effect.

## Results

### Global proteomic analysis of shrimp hemocytes during acute WSSV infection

To understand the global changes triggered by WSSV infection, hemocytes were collected from PBS- and WSSV-injected shrimp at the genome replication stage (12 hpi) and the late stage (24 hpi) of the first WSSV replication cycle [9]. Using a label-free proteomic approach, 868 proteins were identified and quantified. Using a hierarchical clustering algorithm that grouped the shrimp samples by their protein abundance (Fig. S1), we found that WSSV-infected shrimp hemocytes had different proteomic expression patterns at 12 hpi and 24 hpi compared to the corresponding shrimp hemocytes collected from PBS-injected shrimp (Fig. S1A & S1B). No such proteomic clusters were formed by the hemocyte samples collected from PBS-injected shrimp at different time points (Fig. S1C), while two main clusters were formed by the WSSV 12 hpi and WSSV 24 hpi groups (Fig. S1D). Two of the samples, 12-WSSV#1 and 24-WSSV#2, were not assigned to the corresponding cluster, and we therefore excluded these two mis-assigned samples from our subsequent analysis. (We note, however, that even when these two samples are included, the overall protein changes are only very slightly different. Please see Table S1 to compare the results obtained with and without the inclusion of these two anomalous samples).

### Global metabolomic analysis of shrimp hemocytes during acute WSSV infection

**Figure 1:**
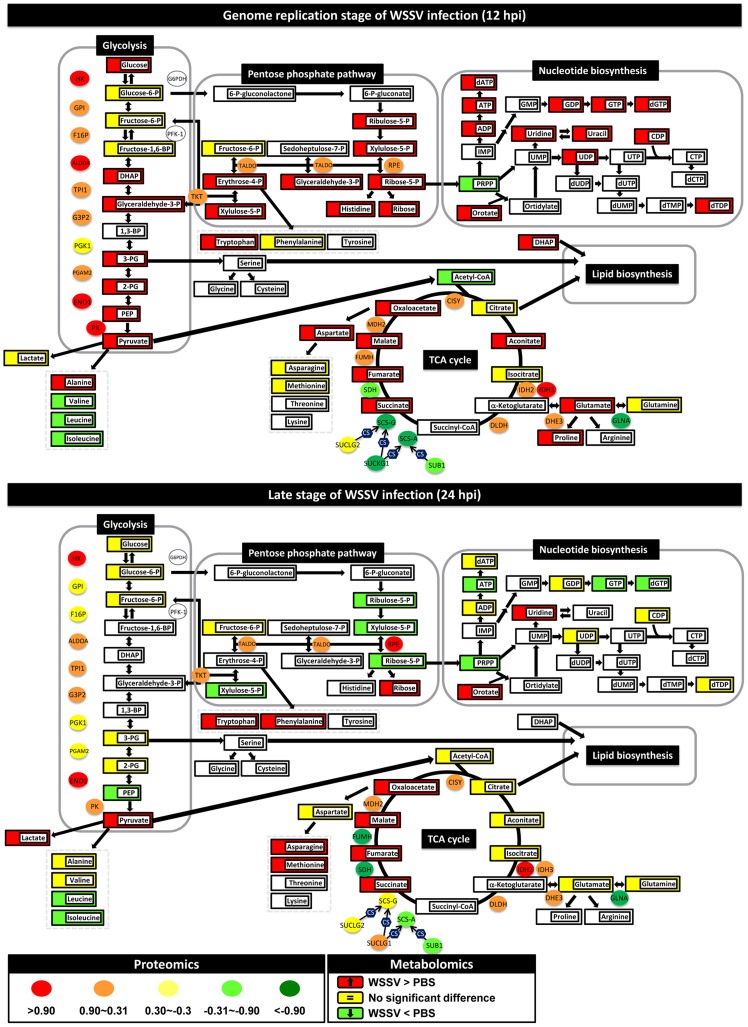
WSSV induces the Warburg effect in the cellular proteome and metabolome of shrimp hemocytes at the replication stage (12 hpi) but not at the late stage (24 hpi). Changes in the levels of enzymes and proteins (ellipses) and metabolites (rectangles) relative to PBS-injected controls are color-coded to represent up- (red) or down- (green) regulation. Yellow represents no change. Colorless boxes and ellipses indicate that no data was detected. Protein data were collected from 3–5 pooled samples of 5 shrimp using quantitative label-free proteomics and expressed on a logarithmic scale. Metabolomic data were collected from 5–6 pooled samples of 10 shrimp using LC-ESI/MS. Numeric values for the proteomic and metabolomic data are given in Tables S1 and S2, respectively.

To further understand the cellular responses after WSSV infection, we also used a global metabolomic platform to measure the metabolic changes in shrimp during WSSV infection. In this study, LC-ESI-MS data on over 100 metabolites were collected at 12 and 24 h after WSSV- or PBS-injection. However, since we were interested primarily in host processes that are involved in the Warburg effect, we focused particularly on a limited number of important host pathways, including glycolysis, the PPP, nucleotide metabolism and the TCA cycle. Our metabolomic and proteomic data are given in Supplementary Tables S1 and S2. Changes in these pathways at 12 and 24 hpi are shown in Figure 1 and are described in more detail below.

### WSSV infection enhances glycolysis at the WSSV genome replication stage (12 hpi)

In mammalian cells, the two main pathways of carbon metabolism, glycolysis and the TCA cycle, oxidize hexose sugars to form ATP and NADPH, or else convert the same sugars to precursors of nucleotides, amino acids, and lipids. In shrimp hemocytes, WSSV infection at 12 hpi has previously been shown to increase glucose consumption and lactate production in ways that resemble the Warburg effect, but details of the intracellular changes in the carbon metabolism have not yet been investigated.

**Figure 2:**
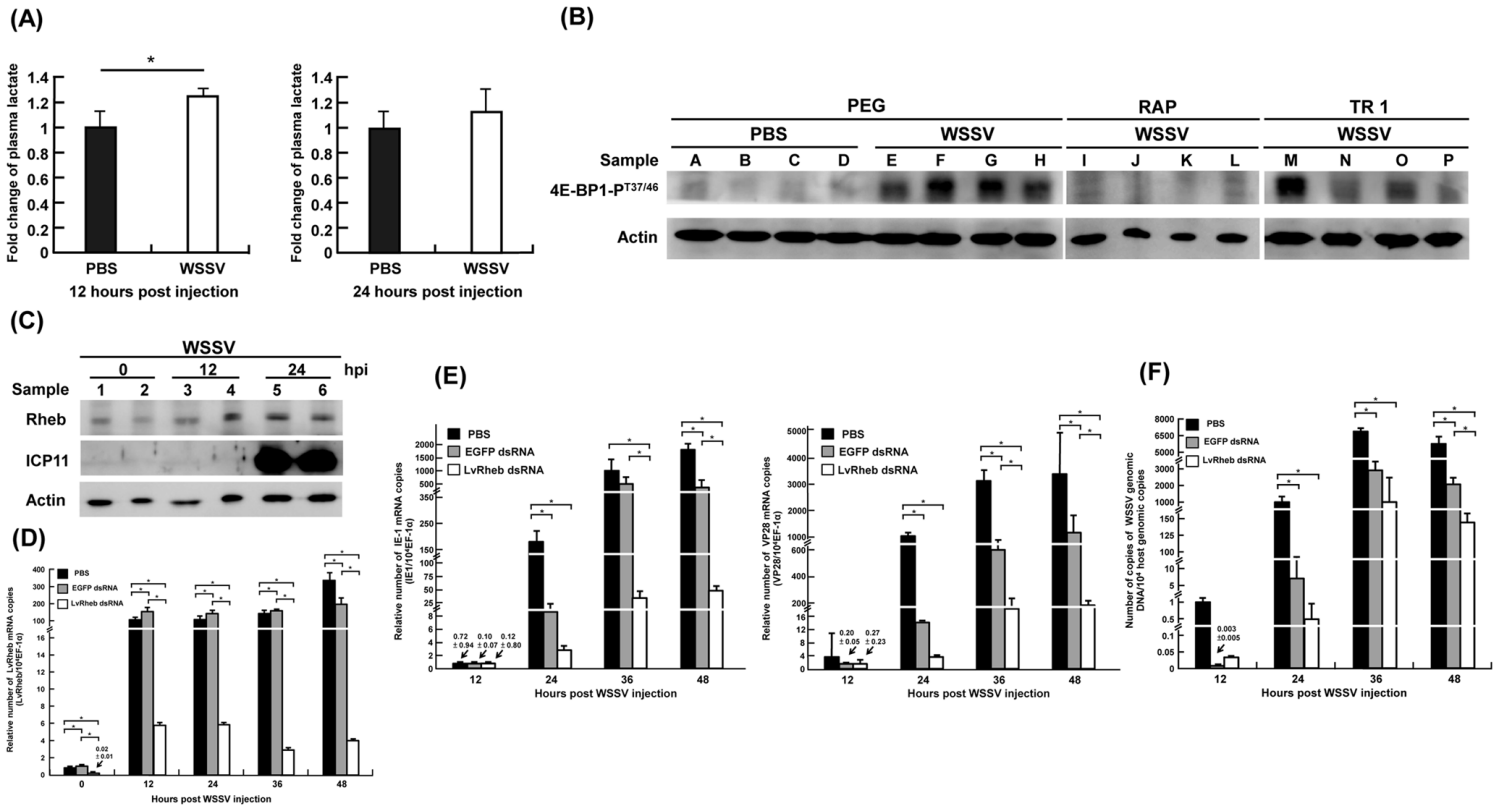
(A) WSSV infection increased the plasma lactate production at the WSSV genome replication stage (12 hpi), rather than at the late stage (24 hpi). The levels of lactate production were profiled and measured by LC-ESI-MS. Each bar represents the mean ± SD from four or five independent samples, with each sample pooled from 10 shrimp. The asterisks indicates a statistically significant difference in lactate production (p<0.05). (B) WSSV-induced phosphorylation of 4E-BP1 was suppressed by treatment with the mTOR inhibitors Rapamycin and Torin 1. Two hours before WSSV injection, shrimp were treated with volume-matched solvent (PEG), Rapamycin (RAP; 0.02 µg/g shrimp) or Torin 1 (TR 1, 25 µg/g shrimp). At 24 h post WSSV injection, twelve shrimp were selected from each group and divided into four sets (A–D, E–H, I–L, M–P). From each set, four pooled samples (3 shrimp in each pool) were prepared by collecting gill samples and extracting the total proteins. Each pooled sample was then subjected to Western blotting with antibodies to phosphorylated 4E-BP1-P^T37/46^ and actin. (C) Shrimp LvRheb is induced after WSSV infection. At the indicated time points after WSSV injection, total protein was extracted from the gills of individual shrimp and subjected to Western blotting. The expression of LvRheb was significantly induced at 24 hpi. ICP11, which is a major WSSV very late protein, was used as a proxy to indicate the WSSV infection state. Actin was used as an internal control. (D) Time series showing that LvRheb dsRNA treatment successfully silenced LvRheb expression in the hemocytes of WSSV-infected shrimp. Three days after LvRheb dsRNA injection, shrimp were injected with WSSV. Hemocytes were collected after the indicated number of days and subjected to cDNA synthesis and real-time PCR. Injections of enhanced green fluorescent protein-dsRNA (EGFP dsRNA) and phosphate-buffered saline (PBS) were used as dsRNA controls. Each bar represents the mean ± SD from four pooled samples with 3 shrimp in each sample. An asterisk indicates a significant statistical difference between groups (p<0.05). (E) Gene silencing of shrimp LvRheb has no significant effect on the expression of the WSSV genes IE1 and VP28 during the first replication cycle (∼24 hpi). The mRNA expression of IE1 gene and VP28 were used as proxies to indicate the WSSV infection state. Each bar represents the mean ± SD from four pooled samples (3 shrimp in each sample). An asterisk indicates a significant statistical difference between groups (p<0.05). (F) Gene silencing of shrimp LvRheb also has no significant effect on the number of WSSV genome copies during the first replication cycle (∼24 hpi). Experimental conditions were as described above. The IQ Real WSSV Quantitative System was used to measure the number of copies of the WSSV genomic DNA. Each bar represents the mean ± SD from four pooled samples (3 shrimp in each sample). An asterisk indicates a significant statistical difference between groups (p<0.05).

In WSSV-injected shrimp hemocytes at 12 hpi, there was a significant increase (p<0.05) in the glycolytic pathway metabolites glucose, dihydroxyacetone phosphate (DHAP), glyceraldehyde-3-P, 3-phosphoglycerate (3-PG), 2-phosphoglycerate (2-PG), phosphoenolpyruvate (PEP) and pyruvate (Fig. 1). There was also a corresponding increase in the protein levels of several glycolytic enzymes (Fig. 1). Despite the increase in glucose uptake, there was no evidence of lactate accumulation at the intracellular level. However, LC-ESI-MS revealed that lactate levels in the hemolymph outside the cells were significantly elevated at this time (Fig. 2A). By contrast, at the late stage of the WSSV replication cycle (24 hpi), except for pyruvate, all of the above-mentioned glycolytic metabolites decreased relative to the PBS-injected control (Fig. 1). Except for hexokinase (HK) and alpha-enolase (ENO1), the associated glycolysis enzymes were also less strongly up-regulated at this time. Meanwhile, there was now a significant accumulation of lactic acid inside the cells, whereas levels in the hemolymph returned to normal (Fig. 2A). Taken together, these results indicate that glycolysis was up-regulated at the WSSV genome replication stage (12 hpi), but not at the late stage of the WSSV replication cycle (24 hpi).

### Apparent re-routing of the glycolytic pathway during WSSV infection

At 12 hpi, we noticed that, even though glycolysis was enhanced, three glycolytic metabolites, glucose-6-P, fructose-6-P and fructose-1,6-BP, remained unchanged relative to the PBS-injected controls (Fig. 1). The reason for this appears to be that glucose-6-P is being rerouted from glycolysis to the first step of the PPP. Although this putative rerouting still needs to be confirmed, e.g. by carbon flux analysis, this would explain why glyceraldehyde 3-P, which is one of the end products of the PPP, was also increased. As Fig. 1 shows, many of the metabolic intermediates in the PPP were strongly up-regulated at 12 hpi even though there was only a relatively weak increase in the protein levels of the corresponding enzymes. However, although the protein level of glucose 6-phosphate dehydrogenase (G6PDH) was not detected in the present study, our previous data shows that there was an increase in this enzyme's activity [9]. This suggests that the putative boost in the PPP at 12 hpi might be driven either by the increase in glycolysis or by changes in the activity of critical enzymes. However, we note that other explanations might also account for the observed accumulation of PPP metabolites, for instance, it is possible that the conversion of ribose-5-P to PRPP (5-phosphoribosyl-1-pyrophosphate) might be blocked.

At 12 hpi, we also observed an apparent boost in nucleotide metabolism, as suggested by increases in the levels of the purine biosynthetic intermediates in ATP synthesis (ADP, ATP, dATP) and dGTP synthesis (GDP, GTP and dGTP) (Fig. 1). Pyrimidine intermediates, including orotate as well as uridine, uracil, UDP, CDP and dTDP, were also up-regulated. This putative boost occurred despite a decrease in the levels of PRPP, presumably because this metabolite, which plays a number of roles in the biosynthesis of purine and pyrimidine nucleotides, was being rapidly consumed. As mentioned above, if conversion from ribose-5-P was in fact blocked, this would also contribute to the reduced levels of PRPP.

All of the above changes would generate building blocks for macromolecular synthesis that could be used by the virus for genome replication. We further note that at 24 hpi, almost all of these increases in the PPP and nucleotide biosynthesis had dissipated.

### WSSV infection disrupts the Tricarboxylic Acid Cycle

The end product of glycolysis, pyruvate, can be further converted into two metabolites, alanine and the important metabolite acetyl-CoA, which occupies a central position between glycolysis, the mitochondrial TCA cycle, beta-oxidation, amino acid biosynthesis and lipid biosynthesis. At 12 hpi, although pyruvate levels were significantly increased, surprisingly there was a decrease in the levels of acetyl-CoA (Fig. 1). The absence of any consistent increase in the levels of citrate, aconitate and isocitrate suggests that the acetyl-CoA was being diverted (via citrate) from the TCA cycle into lipid biosynthesis. At 12 hpi, several other metabolites in the TCA cycle were significantly increased while their respective enzymes were decreased in parallel. A similar disruption was also observed at 24 hpi. Thus for instance, accumulation of the same four metabolites (i.e. succinate, fumarate, malate and oxaloacetate) was observed at both time points. Although these changes might have resulted from a blockage of this pathway, we also note that, at 12 hpi, even though glutamine levels showed no change, glutamate was significantly up-regulated. This suggests an alternative explanation: i.e. that the second half of the TCA cycle is being driven by the conversion of glutamine to α-ketoglutarate via glutaminolysis. Input from glutaminolysis at 12 hpi would also explain why levels of succinate to oxaloacetate were increased even though the protein levels of their respective enzymes were all down-regulated. Lastly we note that both glutaminolysis and partial disruption of the TCA cycle are both well-known consequences of the Warburg effect.

### Enhanced production of amino acid metabolism during WSSV infection

At 12 hpi, WSSV infection induced an increase in several of the detected amino acids, including alanine, histidine, tryptophan, glutamate, proline and aspartate (Fig. 1). At 24 hpi, amino acids were apparently still being synthesized, but the pattern had changed. For example, at 12 hpi, but not at 24 hpi, glutaminolysis seemed to drive the enhanced levels of glutamate, proline and aspartate. Meanwhile, although the high levels of aspartate at 12 hpi were probably being used for *de novo* pyrimidine biosynthesis, by contrast, at 24 hpi, when the amounts of the nucleic acids had returned to control levels, the aspartate was evidently being converted to asparagine and methionine instead. We also note that at 12 hpi, levels of pyruvate and alanine were both elevated, whereas at 24 hpi, even though pyruvate levels somehow remained high (despite the glycolytic pathway mostly shutting down), alanine levels had returned to normal. This appears to be due to the preferential conversion of pyruvate into lactate instead of alanine at this time. Taken together, these data suggest at 12 hpi WSSV infection strongly up-regulates amino acid metabolites that are useful for protein synthesis, and especially those that are involved in glutaminolysis and the synthesis of nucleic acids. This presumably benefits viral genome replication. We note however that this is only a tentative conclusion that still needs to be experimentally tested, and at this time we cannot rule out the possibility that the observed increases in these amino acids are simply due to down regulation or reduced activity of their corresponding enzymes.

### WSSV-induced activation of mTORC1 is not specifically Rheb-dependent

Recent studies have shown that numerous cancer/tumor cells depend on the mTOR signaling pathway to trigger the Warburg effect for efficient cellular proliferation [11]–[13]. Although mTOR is found in two complexes, mTORC1 and mTORC2, only the former is thought to be important for cell growth, proliferation and cellular metabolism [18], [19]. Activation of the mTORC1 pathway causes phosphorylation of its downstream target 4E-BP1, so phosphorylated 4E-BP1 is often used as an indicator of mTORC1 activity [18].

Here we found that the level of phosphorylated 4E-BP1 in pooled samples of shrimp gills was elevated after WSSV injection compared to the PBS controls (Fig. 2B, lanes A–H). Further, when shrimp were injected with the mTORC1 inhibitor Rapamycin (RAP) or the mTORC1/C2 inhibitor Torin 1 (TR1) 2 h before WSSV injection, the WSSV-induced phosphorylation of 4E-BP1 was suppressed in all but one of the pooled samples (Fig. 2B, lanes I–P). From these data, we conclude that WSSV infection results in mTORC1 activation.

Our proteomic data also suggests that the mTORC1 pathway is activated, as shown by the increased expression of several proteins in the mTOR pathway at 12 hpi, including the mTOR activator Rheb (Fig. S2A). We used Western blotting to confirm that Rheb expression was increased in WSSV-infected shrimp at 12 and 24 hpi compared to the control samples at 0 hpi (Fig. 2C).

To further determine if the increase in Rheb expression was important for WSSV replication, shrimp were treated with Rheb dsRNA to silence Rheb expression before being injected with WSSV (Fig. 2D). Although all three groups showed a dramatic increase in Rheb mRNA expression from 0 to 12 hpi, Rheb mRNA expression was still much lower through to 48 hpi in the Rheb dsRNA-treated group compared to the PBS and EGFP dsRNA control groups. However, despite the successful silencing of Rheb expression, during the first WSSV replication cycle (0–24 hpi), the IE1 and VP28 expression levels were only modestly inhibited in the Rheb dsRNA-treated group compared to the EGFP dsRNA-treated group (Fig. 2E), and there was only a correspondingly small decrease in the viral copy number (Fig. 2F). From these results, we concluded that the WSSV-induced activation of the mTOR pathway is partly, but not completely, dependent on Rheb. This conclusion was further supported by Western blots that detected phosphorylated 4E-BP1 after Rheb dsRNA treatment (Fig. S2B), thus showing that mTORC1 activation could still occur even when Rheb was knocked down.

### mTOR activation is critical for the WSSV-induced Warburg-like effect

**Figure 3:**
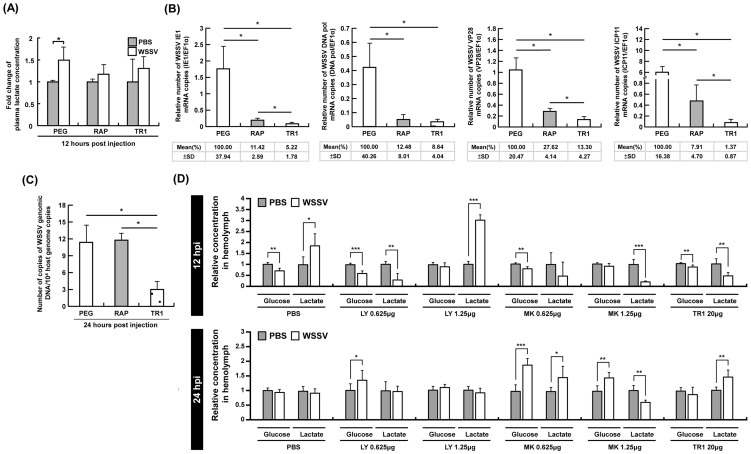
At 12-induced Warburg effect was suppressed by inhibiting mTOR, PI3K and Akt. (A) At 12 h post WSSV injection, the accumulation of plasma lactate that was seen in the PEG-injected control group was suppressed by both Rapamycin (RAP) and Torin 1 (TR1). Each bar represents the mean ± SD from four pooled samples of hemolymph (3 shrimp in each sample). An asterisk indicates a significant statistical difference between groups (p<0.05). The effect of Rapamycin and Torin1 on (B) the expression of the WSSV genes IE1, DNA pol, VP28 and ICP11, and (C) WSSV genome copy number. Data were based on pooled samples of hemocytes (gene expression) or pleopods (genome copy number), with all samples being taken from the same sets of shrimp as those used in Figure 2B. Specifically, the PEG results were from sets E–H, the RAP results from I–L, and the TR1 results from N–P. (Please note that data from the set M was excluded because phosphorylation of 4E-BP1 was not successfully inhibited in this set.). (D) Each bar represents the mean ± SD of the relative glucose or lactate concentration of five pooled samples of hemolymph (3 shrimp in each pooled sample) under the indicated conditions. Statistically significant differences are shown by 1–3 asterisks, which respectively indicate p<0.05, p<0.005 and p<0.0005.

To investigate whether activation of the mTOR pathway might regulate the WSSV-induced Warburg-like effect, we pre-treated shrimp with Rapamycin and Torin 1 to respectively suppress mTORC1- and mTORC1/C2- activation before injection with WSSV. At the WSSV genome replication stage (12 hpi), although lactate levels were elevated in the hemolymph of the non-suppressed WSSV-infected shrimp controls, there was no significant lactate accumulation in WSSV-infected shrimp when mTOR activation was suppressed either by Rapamycin or by Torin 1 (Fig. 3A). Taken together, these data suggest that the mTOR pathway plays an essential role in triggering the Warburg effect.

### In shrimp hemocytes, the mTORC1 pathway is predominant for WSSV gene expression, while inhibition of mTORC1 alone is not sufficient to reduce WSSV replication

Having shown that mTOR activation plays an essential role in triggering the Warburg-like effect during WSSV infection, we next explored what happens to WSSV viral gene expression and viral DNA genome replication when the Warburg-like effect is suppressed by inhibition of the mTOR pathway. As shown in Fig. 3B, both Rapamycin and Torin 1 significantly inhibited the expression of WSSV viral genes in every stage of infection, from IE1, through DNA polymerase (DNA pol), to VP28 and ICP11. Inhibition of the mTORC1 pathway alone was sufficient to account for most of the observed reductions, but the reductions in gene expression were further augmented when the mTORC2 pathway was also suppressed. Although mTOR is known to be important for a range of cellular activities, including the activation of several transcription factors [18], [19], our results are also consistent with the following two ideas: First, that triggering the Warburg-like effect is essential for the successful expression of the WSSV genes and second, that although the mTORC1 and mTORC2 pathways are both involved in triggering the Warburg-like effect, the mTORC1 pathway is predominant. Meanwhile, synthesis of the viral DNA genome was only significantly suppressed by Torin 1 (Fig. 3C). One possible reason for this unexpected result is that blockage of the mTORC1 pathway by Rapamycin does not completely shut down the Warburg effect because it still allows some amount of “leakage” through the mTORC2 pathway. This leakage would also explain the observed significant differences between Rapamycin versus Torin 1 treatment in three of the WSSV gene expressions (IE1, VP28 and ICP11).

### Activation of the upstream PI3K-Akt pathway is also critical for the WSSV-induced Warburg-like effect

Since mTOR activation can be stimulated by the upstream PI3K-Akt pathway [18], we next investigated the effect of inhibiting Akt activation. At 12 hpi, the Warburg effect, which is defined as an increase in both glucose consumption and lactate production, was only seen after pretreatment with PBS (Fig. 3D). (Taken together Fig. 1 and Fig. 3D show that there was an increased uptake of glucose from the hemolymph into the hemocytes.) This WSSV-induced Warburg effect was blocked by pretreatment with MK2206 (an Akt inhibitor). The effect was also blocked by LY294002 (a PI3K inhibitor that also inhibits mTOR) and by Torin 1. There was no evidence of a Warburg effect at 24 hpi under any treatment condition (Fig. 3D). In a separate experiment we also found that pretreatment with LY294002 suppressed phosphorylation of 4E-BP1 at 24 h after WSSV infection (Fig. S2C).

### WSSV replication depends on the PI3K-Akt-mTOR pathway

**Figure 4:**
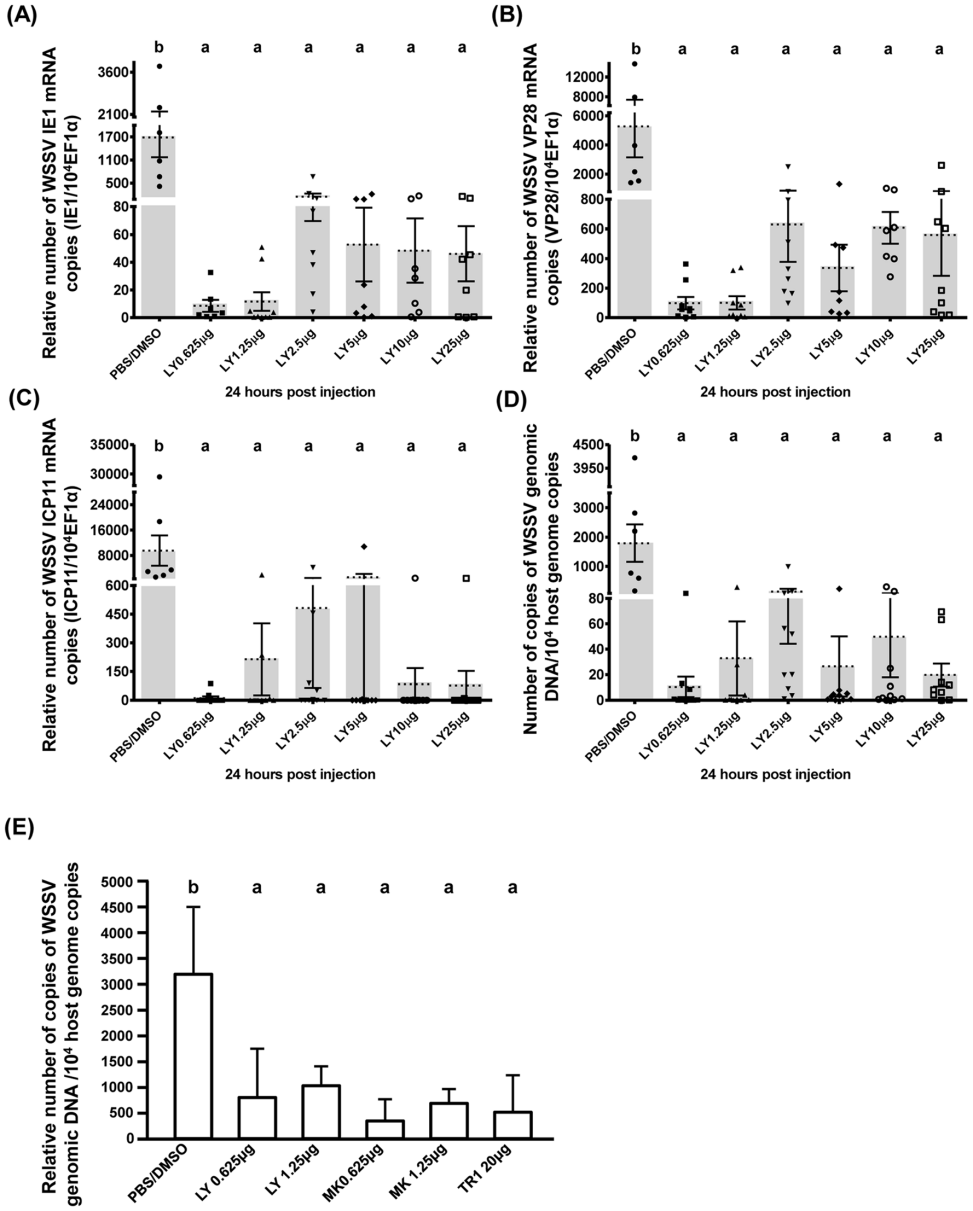
WSSV replication was suppressed when the PI3K-Akt-mTOR pathway was inhibited by LY294002, MK2206 and Torin 1. Shrimps were pretreated with LY294002 and samples were collected at 24(A–D) show the expression of WSSV (A) IE1, (B) VP28, and (C) ICP11 in shrimp hemocytes and (D) the viral copy number in pleopods from the same groups of LY294002-pretreated shrimp (n = 6–10 in each group). Graph (E) shows the mean WSSV copy number in five pooled samples of pleopods (3 shrimp in each pooled sample) collected from another batch of experimentally infected shrimp after pretreatment with the indicated inhibitor. Bars labeled with different letters indicate significantly different values (p<0.05).

To determine if the PI3K-Akt-mTOR pathway is critical for the promotion of WSSV gene expression and genome replication, we investigated the effect of the three inhibitors, LY294002, MK2206, and Torin 1. As shown in Fig. 4, treatment with 0.625–25 µg of LY294002 per g of shrimp body weight led to a significant decrease in WSSV gene expression (Fig. 4A–C) and WSSV DNA genome replication (Fig. 4D). A second experiment with another batch of shrimp further showed that treatment with LY294002, MK2206 and Torin 1 led to significant and equal reductions in the WSSV genome copy number (Fig. 4E). These results suggest that WSSV infection may trigger the Warburg-like effect by activating the PI3K-Akt-mTOR pathway.

To investigate the role of PI3K only, we used the selective pan-class I PI3K inhibitor BKM120. Although we were only able to conduct a pilot study, our results show that pretreatment with 0.625 µg BKM120 per g shrimp significantly reduced WSSV copy number (Fig. S2D). This provides further evidence that activation of the PI3K-Akt-mTOR pathway is important for WSSV replication.

### Inhibition of mTOR activation by Torin 1 prevented the WSSV-induced Warburg effect in shrimp hemocytes

**Figure 5:**
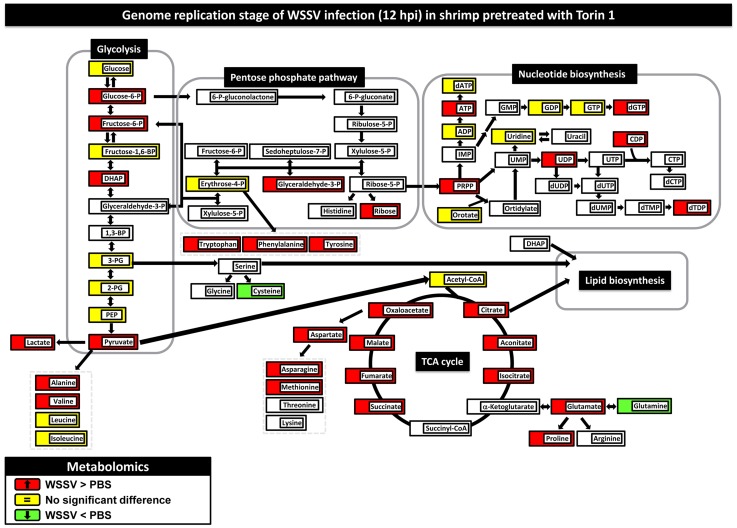
Pretreatment with Torin 1 inhibited the WSSV-induced Warburg effect at the genome replication stage of WSSV infection (12 hpi) in shrimp hemocytes. Two hours after treatment with Torin 1, shrimp were injected with PBS or a WSSV inoculum. At 12(10 shrimp per pool) were collected from each group. Changes in the metabolomic levels of the WSSV-infected samples relative to the PBS controls are color-coded as described in Figure 1. Figure S3 shows changes in the metabolome at 24 hpi. Numerical data for 12 hpi and 24 hpi is given in Table S2. Changes in the metabolome for Torin-PBS versus PEG-PBS are shown in Fig. S3, with numerical data given in Table S2.

To investigate whether the decrease in WSSV gene expression and viral genome copies in Torin 1-pretreated shrimp (Figs. 3 & 4) might be due to cancellation of the Warburg-like metabolic changes, we compared the metabolic profiles of WSSV-infected hemocytes at 12 hpi either with or without Torin 1 treatment. Figure 5 shows that when shrimp were pretreated with Torin 1, the WSSV-induced Warburg-like effect was no longer observed at 12 hpi (cf. Fig. 1 and Fig. 5). In particular, in the glycolytic pathway, glucose was no longer accumulated, while the accumulation of glucose-6-P suggests that there was no rerouting into the PPP. Unlike Figure 1, Figure 5 shows no universal increase in nucleic acid synthesis, and this is also consistent with the observed accumulation of PRPP. Intracellular levels of lactate also increased. Meanwhile although the TCA cycle and amino acid biosynthesis were both up-regulated, the decrease in WSSV gene expression (Fig. 3) suggests that these changes alone are insufficient for WSSV pathogenesis. In the Torin 1-pretreated shrimp, there was also no evidence of any WSSV-induced Warburg-like metabolic changes at 24 hpi (Fig. S3A). In control experiments where Torin 1-treated shrimps were injected with PBS, at 12–24 hpi the metabolic intermediates were either down-regulated or remained unchanged (Fig. S3B, Table S2).

## Discussion

Although only a limited number of vertebrate viruses (HCMV, HCV, Dengue virus, influenza virus, HSV and KSHV) have been studied using a systems biology approach [5], [6], [8], [20]–[23], in the present study, several metabolic pathways that are often altered in cancer/tumor cells and virus-infected vertebrate cells also seemed to be similarly affected by WSSV infection (Fig. 1 and Tables S1, S2). These altered metabolic pathways included glycolysis, the PPP, the biosynthesis of nucleic and amino acids, and the TCA cycle.

The hallmark of the Warburg effect is aerobic glycolysis, i.e. a high rate of glycolysis accompanied by lactic acid production, despite readily available oxygen [3]. The effect is induced by several vertebrate oncogenic viruses in cultured mammalian cells [13], and in vertebrate cells infected with viruses such as HCMV [6]. Similarly, at 12 h post WSSV infection, i.e. the genome replication stage, glycolytic enzymes and intermediates were strikingly elevated and glucose uptake and lactate excretion were both increased (Figs. 1, 2A and 3D). In vertebrates, glucose transporter modulates this increased glucose uptake [24]; similarly, WSSV proteins interacted with glucose transporter during WSSV infection [25].

We previously reported that the activity of G6PDH was increased at the WSSV genome replication stage [9]. In the present study hexokinase (HK) levels were also increased (Fig. 1); therefore, we inferred that glucose was being rapidly converted into glycolytic intermediates. However, in the absence of an effect on the levels of glucose-6-P (its immediate downstream metabolite), we suggested that the glycolytic pathway was being rerouted into the PPP. Although we still lack carbon flux analysis data to show this directly, our present observations of significant increases in PPP intermediates and compounds used for nucleotide biosynthesis now provides further evidence that WSSV does in fact induced up-regulation of the PPP.

In cancer cells, the PPP is usually up-regulated to balance cellular redox conditions and to ensure an adequate supply of ribose-5-phosphate (R5P) for nucleotide synthesis, a rate-limiting step in cancer cell proliferation [26], [27]. Similarly, viral replication requires biosynthetic precursors supplied by host cell metabolism, and synthesis of viral DNA creates a great demand for DNA precursors to be supplied through salvage reactions or *de novo* synthesis [22], [28], [29]. To ensure an adequate supply of nucleotides, viruses use several strategies, including expressing virus-encoded enzymes that boost nucleotide synthesis, and increasing the flux of the PPP [6], [8], [21], [23], [29], [30]. With its large DNA genome (∼307 kbp) and rapid replication cycle (22–24 h/cycle), WSSV appears to use both strategies. The WSSV genome encodes several viral enzymes related to nucleotide synthesis, e.g., dUTPase, thymidine kinase-thymidylate kinase (TK-TMK), and the ribonucleotide reductase subunits RR1 and RR2, all of which are implicated in modulation of host nucleotide synthesis during WSSV infection [31]. We found that the PPP intermediates and the downstream nucleotides were up-regulated only at the WSSV genome replication stage (12 hpi) (Fig. 1). Even through WSSV induced up-regulation of the host's PPP enzymes at both 12 and 24 hpi (Fig. 1), we reported that the activity of Glucose-6-phosphate dehydrogenase (G6PD), the rate-limiting enzyme of the pentose phosphate pathway, was increased at 12 hpi [9]. We therefore inferred that the putative increase in PPP activity was mainly being driven not only by increased glycolysis, but also by enzyme activity during WSSV infection. Furthermore, we speculate that the observed down-regulation of PRPP (Fig. 1) occurred for different reasons at different times. At 12 hpi, the putatively increased throughput of both the PPP and nucleotide synthesis pathways would result in a high demand for PRPP. This would lead to low levels of PRPP, while conversely, high levels of this intermediate at 24 hpi could be explained by these two pathways shutting down. However, this explanation of the observed changes will need to be confirmed by further studies.

WSSV also induced an increase in the levels of the free amino acids, most of which are either derived from TCA cycle intermediates or converted from pyruvate and glutamine. Mazurek *et al* [32] reported that the increased production of amino acids during HPV infection resulted from both aerobic glycolysis and glutaminolysis. WSSV has already been shown to induce aerobic glycolysis, and Figure 1 now provides evidence that glutaminolysis is also induced: the levels of glutamate and its immediate downstream product, proline, were both up-regulated at the WSSV genome replication stage, while glutaminolysis would also explain the observed replenishment of the partially halted TCA cycle, and thus the increased levels of oxaloacetate and aspartate. Taken together, all of these results suggest that WSSV infection regulates both of these pathways to enhance the production of amino acids.

Acetyl-CoA, which can be converted from pyruvate, occupies a nodal position at the bifurcation of the anabolic and catabolic pathways [33], and it is involved in glycolysis, the TCA cycle, generation of nucleic acids and lipid metabolism. Biosynthesis in cancer cells consumes enormous amounts of energy and acetyl-CoA, resulting in a shortage of the latter for catabolic oxidative processes [33]. In cancer cells under the Warburg effect, shortages of acetyl-CoA also result from a hypoxia-inducible factor (HIF)-1-mediated mechanism that prevents pyruvate dehydrogenase (PDH) from catalyzing the conversion of pyruvate to acetyl-CoA [2]. In contrast, Munger *et al.* [5] reported a dramatic increase in acetyl-CoA during the Warburg effect in HCMV-infected cells. However, in the present study, there was a significant decrease of acetyl-CoA at 12 hpi, but no increase in citric acid levels (Fig. 1). Therefore, we concluded that the WSSV-induced Warburg effect consistently resembled the Warburg effect in cancer cells, although there were some differences from the Warburg effect induced by other viruses.

With such a marked decrease in the amount of acetyl-CoA entering the TCA cycle, it was surprising to see an accumulation of the TCA cycle intermediates succinate, fumarate, malate and oxaloacetate at 12 and 24 hpi. Two possible mechanisms might account for this. First, in proliferating glioblastoma cells, glutaminolysis, which ultimately results in the conversion of glutamine to lactate, provides an anaplerotic source for the TCA cycle and allows it to produce enough NADPH to support fatty acid synthesis [34]. Since glutaminolysis was also apparently up-regulated in the WSSV-infected hemocytes at 12 hpi (Fig. 1), this might likewise serve to replenish the TCA cycle and explain the observed accumulation of the downstream TCA cycle intermediates. Another possible reason for the accumulation of these metabolites may be the down-regulation of related enzymes in the TCA cycle, such as succinyl-CoA synthetase (SCS), succinate dehydrogenase (SDH) and fumarate hydratase (FUMH) (Fig. 1). Recent studies suggest that SDH and FUMH behave as classic tumor suppressors [2]. When these enzymes are down-regulated, their respective substrates, i.e. succinate and fumarate, accumulate in the mitochondria and leak out into the cytosol, where their presence leads to an inhibition of prolyl hydroxylase enzymes (PHDs). Inhibition of the PHDs allows the activation of HIF, which in turn causes pseudo-hypoxia and enhances both neovascularization and glycolysis within the cell [35]. Both glutaminolysis and enzyme reduction may be important at 12 hpi, but as Figure 1 suggests, the downregulation of SCS, SDH and FUMH may be more important at 24 hpi because glutaminolysis has apparently returned to approximately baseline levels at this time.

The phosphorylation of 4E-BP1 (Fig. 2B) shows that in the absence of the inhibitors Rapamycin and Torin 1, WSSV infection activates the PI3K-Akt-mTOR pathway. Meanwhile, as shown by Figure 3A and Figure 5, pretreatment with the mTOR inhibitor Torin 1 disrupts all of the above WSSV-induced metabolic changes. Taken together, these results suggest that the WSSV-induced Warburg effect is being driven by activation of the PI3K-Akt-mTOR pathway. This pathway is known to trigger the Warburg effect in cancer cells [11]–[13], and it is also used by human papillomavirus to trigger the Warburg effect and achieve successful replication [4], [36].

When the Warburg effect was shut down by using PI3K-Akt-mTOR inhibitors, viral gene expression and viral copy number were significantly reduced (Figs. 3 & 4). We note that even though the TCA cycle was up-regulated in the Torin 1 pretreated shrimp at 12 hpi (Fig. 5), the expression of viral genes and viral copy numbers was still repressed (Fig. 3). We infer from this that even when the normal source of energy (i.e. the TCA cycle) is activated, it still fails to provide sufficient energy and materials to meet the demands of the virus, and that these requirements can only be satisfied by shifting the metabolism into aerobic glycolysis.

Our results show that when the PI3K-Akt-mTOR pathway is entirely blocked, i.e. by either an upstream inhibitor of PI3K or Akt (Fig. 4 and Fig. S2D) or by Torin 1 (Fig. 3), viral copy number is significantly reduced. We show here that the Warburg effect is also important for WSSV replication. However, we unexpectedly found that although Rapamycin was able to block the Warburg effect and the expression of viral genes (Fig. 3A, B), it did not have any significant impact on virus genome copy number (Fig. 3C). We hypothesize that these apparently inconsistent observations may be due to the mTORC2 pathway, which is somehow able to provide an alternative route when mTORC1 is blocked by Rapamycin. It would be interesting to see if a similar result could be produced by blocking mTORC2 only; unfortunately our attempts to achieve this with dsRNA silencing have so far been unsuccessful.

Interestingly, silencing Rheb, which acts as a positive regulator of mTORC1 signaling, does not significantly affect virus propagation during the first replication cycle (Fig. 2D–F). Two possible mechanisms might account for this. First, during WSSV infection, mTORC1 activation might not depend exclusively on Rheb. Some vertebrate viruses, such as HCMV, can modulate the PI3K-Akt-mTOR signaling pathway via a surprisingly large number of alternative viral proteins [37]–[41]. It therefore seems possible that WSSV might likewise express one or more viral proteins that functionally replace the mTOR activator Rheb or otherwise activate the mTOR pathway. The second possibility is that mTORC2 can, to some extent, act to compensate for the suppression of mTORC1. As shown in Figure 3A, mTORC1 is essential for inducing the Warburg effect, and it is also predominant in viral gene expression (Fig. 3B). Nevertheless, successful viral genome replication can be achieved by mTORC2 alone (Fig. 3C). Although these two mTOR complexes normally differ in their substrate specificity and function, during HCMV infection, mTORC2 becomes involved in the phosphorylation of the mTORC1 substrates 4E-BP1 and S6K [39]. Kudchodkar *et al.* [39] proposed that this specific alteration was due to structural modification caused by the addition of viral or cellular regulatory proteins to the mTOR complexes. If something similar is happening in WSSV, this suggests that there must still be other important WSSV proteins involved in the modulation of the PI3K-Akt-mTOR signaling pathway that remain to be discovered.

**Figure 6:**
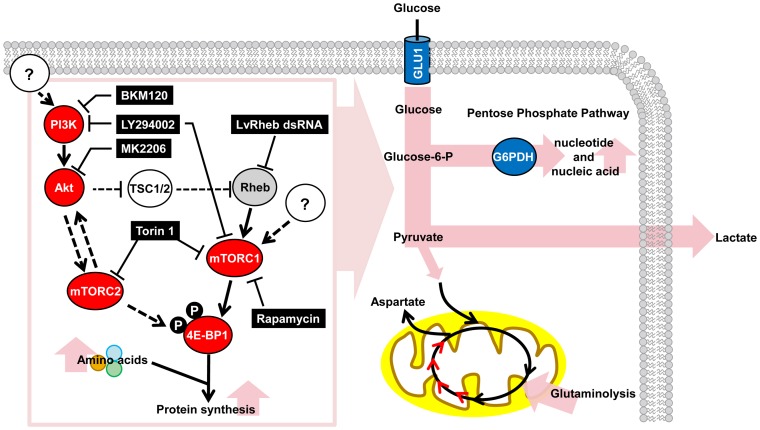
Schematic representation of the WSSV-induced Warburg effect and the involvement of the PI3K-Akt-mTOR pathway at the viral genome replication stage (12 hpi) of the first WSSV replication cycle. For the proteins investigated in this study, red indicates a positive involvement and gray indicates a partial involvement. Blue indicates important upregulated proteins and intermediates from previous studies: Glucose transporter (GLU1) is from Huang *et al.* [25], and G6PDH is from Chen *et al.* [9]. Elevated metabolic pathways are shown in pink. Pathway inhibitors are indicated in black boxes. Dashed lines indicate inferred regulatory mechanisms that were not investigated in the present study.

In this study, in addition to providing a global metabolic view of the invertebrate Warburg effect during WSSV infection, we have developed a model of how WSSV may trigger these metabolic changes. A schematic is presented in Figure 6. In this model, the PI3K-Akt-mTOR pathway is activated after WSSV infection, and at the WSSV genome replication stage (12 hpi), the mTOR complexes mTORC1 and mTORC2 are both involved. The Warburg effect and the anaplerotic effect of glutaminolysis both benefit the virus by increasing the availability of the building blocks and energy necessary to meet WSSV's requirements during genome replication. While it will be important to verify this model using techniques such as carbon flux analysis, which might be conveniently carried out in the relatively long-lived hematopoietic cells of an *in vitro* crayfish culture [42], another interesting subject for future study will be to identify the putative viral factors that are involved in the activation of the PI3K-Akt-mTOR pathway.

## Materials and Methods

### Virus inoculum and experimental animals

The virus used in this study was the WSSV Taiwan isolate. To prepare the WSSV stock, hemolymph was extracted from WSSV-infected moribund shrimp and subjected to centrifugation (10,000×g). The supernatant was then diluted with phosphate-buffered saline (PBS) (137 mM NaCl, 2.7 mM KCl, 10 mM Na_2_HPO_4_, 2 mM KH_2_PO_4_), and stored at −80°C. The experimental inoculum was prepared from this stock by dilution (10^−4^) with PBS. *Litopenaeus vannamei* shrimp (mean weight: 3–5 g) were obtained from the Aquatic Animal Center at National Taiwan Ocean University. Before the experiments, shrimp were cultured in a water tank system containing filtered seawater (30 ppm at 25 to 27°C) for 1–3 days. In the WSSV challenge experiments, the shrimp were challenged with WSSV inoculum (100 µl/shrimp) by intramuscular injection.

### Ethic statement

All of the shrimp used in this study were obtained from the Aquatic Animal Center at National Taiwan Ocean University. These animals were specifically raised for research purposes and Taiwan does not require any additional permit or permission. Because the experimental animals were invertebrates, no specific permits were required for this study, and there is no official recommendation for the use of shrimp for scientific purposes in Taiwan. Nevertheless all of our experimental procedures, including animal sacrifice, were designed to be as humane as possible, and all animals were treated so as to minimize suffering at all times.

### Antibodies

Primary antibodies used in this study include phospho-4E-BP1 (Thr37/46) (Cell Signaling; Catalog No. 2855), Rheb (Cell Signaling; Catalog No. 4935), and Actin (Millipore). The antibody that recognizes the major WSSV late protein ICP11 was prepared in the lab as described previously [43].

### Preparation of the mTOR inhibitors Rapamycin and Torin 1

Rapamycin (sirolimus) stock was prepared by dissolving Rapamycin powder (Sigma-Aldrich Co.) in 99% ethyl alcohol. Before use, this stock was diluted with PEG solvent (0.25% polyethylene glycol, 0.25% Tween 20 and 0.15 M NaCl). Torin 1 (Tocris Bioscience) was dissolved in dimethyl sulfoxide (DMSO) to provide a stock solution. Before use, this stock was diluted with PEG solvent.

### Effect of Rapamycin and Torin 1 on 4E-BP1 phosphorylation, plasma lactate, viral genes and virus copy number

To evaluate the involvement of the mTOR complexes during WSSV infection, shrimp were pretreated with Rapamycin (0.02 µg/g shrimp) or Torin 1 (20 µg/g shrimp) by intramuscular injection 2 h before being challenged. Control shrimps were injected with PEG only. At 12 and 24 h after the pretreated shrimps were injected with WSSV or PBS, four pooled samples of gills, hemolymph, hemocytes, and pleopods were collected from each group with each pooled sample being taken from 3 shrimp. Western blotting was used to measure the protein levels of phospho-4E-BP1 in the gills. Lactate levels in the hemolymph were measured as described below. Real-time PCR was used to measure virus gene mRNA expression and virus copy number. See below for details of these procedures.

### Effect of the PI3K-Akt-mTOR inhibitors LY294002, MK2206 and BKM120

To evaluate the involvement of the PI3K-Akt-mTOR pathway during WSSV infection, shrimp were pretreated with inhibitors LY294002 (0.625–1.25 µg LY294002/g shrimp), MK2206 (0.625–1.25 µg MK2206/g shrimp) and BKM120 (buparlisib; 0.15625–25 µg/g shrimp) by intramuscular injection 2 h before being challenged. Stock solutions were prepared by dissolving LY294002 (Biovision), MK2206 (Biovision) and BKM120 (Selleckchem) in 10% DMSO, and these solutions were further diluted with PBS before use. Control shrimps were injected with 0.01% DMSO in PBS. At 24 h after the pretreated shrimps were injected with WSSV, hemocyte and pleopods samples were collected from 6–10 individual shrimp in each group. The hemocyte samples were subjected to real-time PCR to measure the mRNA levels of WSSV IE1, VP28 and ICP11 as described below. Real-time PCR was also used to measure the viral copy number in the pleopods samples.

### Label-free quantitative proteomic analysis of the shrimp hemocyte by liquid chromatography tandem mass spectrometry (LC-MS/MS)

At 12 and 24 h after shrimp were injected with PBS or WSSV, 4–5 pooled hemocyte samples (5 shrimp in each sample) were collected from each group using an anticoagulant (450 mM NaCl, 10 mM KCl, 10 mM EDTA, 10 mM Tris-HCl, pH 7.5). After centrifugation at 10,000×g for 1 min followed by washing twice with 1× PBS, hemocytes were resuspended with 0.25× PBS to extract total protein for LC-MS/MS based label-free quantitative proteomic analysis. The total protein content of the lysates was quantified using a Bradford protein assay kit (Bio-Rad) with bovine serum albumin (BSA) added as an internal quantitative standard for each analysis. The lyophilized hemocyte protein lysate (60 µg) was resolubilized in an 8 M urea/25 mM ammonium bicarbonate buffer, incubated for 1 h at 37°C with 2 mM dithioerythreitol, and alkylated for 1 h using 2.5 mM iodoacetamide at room temperature. Each sample was then diluted with 25 mM ammonium bicarbonate to a final urea concentration of 1 M and Trypsin (Promega) was added (1∶50 w/w). After overnight incubation, protease activity was quenched by acidification of the reaction mixture with formic acid solution (pH 1–2). Aliquots of the peptide mixtures were desalted and concentrated on a C_18_-StageTip (Proxeon Biosystems), and then eluted with 50% acetonitrile in 0.1% formic acid.

The resulting peptide mixtures were analyzed by online nanoflow liquid chromatography tandem mass spectrometry (LC-MS/MS) on a nanoAcquity system (Waters, Milford, MA) connected to an LTQ Orbitrap Velos hybrid mass spectrometer (Thermo Fisher Scientific, Bremen, Germany) equipped with a PicoView nanospray interface (New Objective, Woburn, MA). After loading onto a 75-µm×250-mm nanoACQUITY UPLC BEH130 column packed with C18 resin (Waters, Milford USA), the peptide mixtures were separated at a flow rate of 300 nl/min using a linear gradient from 5% to 40% solvent B (acetonitrile with 0.1% formic acid) for 90 min. The LTQ Orbitrap Velos instrument was operated in standard data-dependent acquisition mode, automatically switching between full-scan MS and CID (collision induced dissociation)-MS/MS acquisition.

For the CID-MS/MS top20 method, full scan MS spectra (from m/z 350–1600) were acquired in the Orbitrap analyzer at a resolution of 60,000 (at 400 m/z) and an AGC (automatic gain control) target value of 10^6^. The 20 most intense peptide ions with charge states ≥2 were sequentially isolated to a target value of 5,000 and fragmented in the ion trap at 35% normalized collision energy, with an activation q of 0.25, 10 ms activation time, and minimum ion selection intensity of 500 counts.

Progenesis LC-MS (Nonlinear Dynamics, version 3.0) was used for label-free quantification analysis. All raw spectral files were first aligned and only high quality peptide features (charge state>1 and isotopic pattern> = 3) were used for the extraction of ion intensity data from the MS1 spectra. To adjust for system errors such as sample loading and intensity shift across LC-MS/MS runs, the peptide ion intensity was normalized using the Progenesis LC-MS robust mean, which was derived from the peptide log2 ratio distributions between a reference and the targeted LC-MS/MS run. The peak list generated from the qualified peptide features was used to search against a combined database that consisted of an in-house white shrimp database, the shrimp white spot syndrome virus database from NCBI, and the common Repository of Adventitious Proteins database downloaded from the Global Proteome Machine in the MASCOT 2.3 server (Matrix Science). To keep the false discovery rate below than 5% (as estimated from the target-decoy database), only peptides with a Mascot ion score greater than 17 were included for subsequent protein analysis. Proteins were automatically assigned to functional group, and only these proteins that met both of the following criteria were reported: 1) the protein had the most peptide hits within its group, and 2) the protein included at least one unique, quantifiable peptide. Protein quantitation was based on the sum of the total ion intensity of the unique peptides. Lastly, to map the results into the biological network, MetaCore network software (GeneGO) was used for pathway analysis of the expressed proteins.

### Using liquid chromatography electrospray ionization mass spectrometry (LC-ESI-MS) to monitor the effect of Torin 1 on the metabolome of WSSV-infected shrimp

In this experiment, 2 h before shrimp were challenged with WSSV or PBS, they were pretreated with PEG or Torin 1 (20 µg/g shrimp) by intramuscular injection to produce a total of four experimental groups: the PEG-PBS group, the PEG-WSSV group, the Torin 1-PBS group and the Torin 1-WSSV group. At 12 and 24 hpi, 5–6 pooled hemocyte samples (10 shrimp in each sample) were collected from each group using anticoagulant as described above. After centrifugation at 800×g for 1 min followed by washing twice with 1× PBS, the hemocytes were resuspended with 0.33× PBS and kept on ice for 10 min. The samples were then centrifuged at 10,000×g for 10 min, and 100% MeOH was added to the supernatant at a ratio 1∶ 2. After being centrifuged again at 10,000×g for 10 min, the supernatants were lyophilized, dissolved in 35 µl ddH2O and subjected to LC-ESI/MS metabolomic analysis as follows:

To enhance the detection of the carboxylic acid and organic phosphate signals, 5 µl aniline/HCl reaction buffer (0.3 M aniline [Sigma-Aldrich, USA] in 60 mM HCl) and 5 µl of 20 mg/ml N - (3-dimethylaminopropyl)-N′-ethylcarbodiimide hydrochloride (EDC; Sigma-Aldrich, USA) were added to each sample of the hemocyte residue. Each mixture was vortexed and incubated at 25°C for 2 h, after which the reaction was stopped by adding 5 µl of 10% ammonium hydroxide. The aniline derivatized samples were then analyzed using an LC-ESI-MS system consisting of an ultra-performance liquid chromatography (UPLC) system (Ultimate3000 RSLC, Dionex) and a quadrupole time-of-flight (TOF) mass spectrometer with an the electrospray ionization (ESI) source (maXis UHR-QToF system, Bruker Daltonics). The shrimp metabolites were separated by reversed-phase liquid chromatography (RPLC) on a BEH C_18_ column (2.1×100 mm, Walters). The LC parameters were as follows: autosampler temperature, 4°C; injection volume, 10 µl; and flow rate, 0.4 ml/min. After pre-starting with 1% mobile phase B (0.1% formic acid in ACN) for 4 min, the elution started from 99% mobile phase A (0.1% formic acid in ddH_2_O) and 1% mobile phase B (0.1% formic acid in ACN). After holding at 1% for 0.5 min and raising to 60% over 5 min, mobile phase B was further raised to 90% in another 0.5 min, held at 90% for 1.5 min, and then lowered back to 1% in 0.5 min. The column was then equilibrated by pumping 99% B for 4 min. The acquisition parameters for LC-ESI-MS chromatograms were as follows: dry gas temperature, 190°C; dry gas flow rate, 8 L/min; nebulizer gas, 1.4 bar and capillary voltage, 3,500 V. Mass spectra were recorded from m/z 100–1000 in the negative ion mode. Data were acquired by HyStar and micrOTOF control software (Bruker Daltonics) and processed by DataAnalysis and TargetAnalysis software (Bruker Daltonics). Each metabolite was identified by matching with its theoretical m/z value and with the isotope pattern derived from its chemical formula. The identified metabolites were quantified by summing the corresponding area of the extracted ion chromatogram, and metabolite signal levels were presented as the mean of the 5–6 pooled hemocyte samples from each experimental group at each time point.

To investigate the WSSV-induced metabolic changes in shrimp hemocytes, the fold changes in the PEG-WSSV group were calculated relative to the PBS injection group (PEG-PBS group). To investigate the WSSV-induced metabolic changes in the mTOR-inactivated shrimp, the fold changes in the Torin 1-WSSV group were calculated relative to the PBS injection group (Torin 1-PBS group). Lastly, the effect of Torin 1 pretreatment was shown by calculating the fold changes of the Torin 1-PBS group relative to the PEG pretreatment group (PEG-PBS group). Student's *t*-test was used to identify statistically significant changes.

### *In vivo* knock-down of LvRheb expression by dsRNA-mediated RNA interference

Preparation of the dsRNA was done following Wang *et al* [44]. Briefly, first, the partial sequences (approximately 300–400 bp) of LvRheb and EGFP were generated and amplified by PCR for use as linearizing DNA templates. Next, the T7 promoter sequence was incorporated into these linearized DNA templates by using PCR with the following specific primer sets: Experimental group: LvRheb-dsT7F531/LvRheb-R882 and LvRheb-F531/LvRheb-dsT7R882; Control group: EGFP-dsT7F/EGFP-dsR and EGFP-dsT7R/EGFP-dsF (see Table S3 for details). The T7 RiboMAX Express large-scale RNA production system (Promega) was then used to synthesize the ssRNAs according to the manufacturer's instructions. The corresponding ssRNAs were mixed and annealed to become dsRNA by incubation at 70°C for 20 min, followed by slowly cooling to room temperature for 30 min. After purification and precipitation of the dsRNA by phenol/chloroform/isoamyl alcohol extraction, the dsRNA were quantified by UV spectrophotometer and verified by agarose gel electrophoresis. The final dsRNA products were stored at −80°C before being used in the following *in vivo* experiments.

For the gene silencing experiments, the experimental group was injected with LvRheb dsRNA (1 µg/g shrimp), while the control groups were injected with EGFP dsRNA or PBS only. To determine the efficiency of the gene silencing for pooled hemocytes samples (3 shrimp in each pool sample) were collected from each group at the indicated time points. Total RNA was extracted from these samples, and cDNA was synthesized using Superscriptase II Reverse Transcriptase (Invitrogen) with Anchor-dTv primer (Table S3). Real-time PCRs were then performed to measure the expression levels of LvRheb and EF1-α with the following specific primer sets: LvRheb-qF/LvRheb-qR and EF1-α-qF/EF1-α-qR. In uninfected shrimp, gene silencing was maximally effective at 3 days after dsRNA injection (data not shown). In subsequent experiments, the shrimp were therefore challenged at 3 days post dsRNA injection.

### Quantification of WSSV gene (IE1 and VP28) expression in LvRheb-knockdown shrimp

For this knockdown experiment, shrimp were randomly divided into 3 groups and injected with LvRheb dsRNA, EFGP dsRNA, or PBS. At 3 days post dsRNA injection, shrimp were then challenged with WSSV. Four pooled hemocyte samples were collected from each group at various time points (12, 24, 36, and 48 hpi), with each pooled sample taken from 3 shrimp. Total cDNA was then prepared from each sample as described above. To quantify the relative expression of the WSSV *ie1* and *vp28* genes, real-time PCR was performed with the specific primers IE1-qF/IE1-qR, VP28-qF/VP28-qR, and EF1-α-qF/EF1-α-qR (Table S3) using the Bio-Rad detection system with Brilliant SYBR Green QPCR master mix (Applied Biosystems). Data values were calculated by the 2^−ΔΔCT^ method. Statistically significant differences between groups were analyzed by Student's *t*-test.

### Quantification of the WSSV genome copy number in LvRheb-knockdown shrimp

Four pleopod samples (3 shrimp in each sample) were also collected from of the above experimental groups at the same time points. The samples were subjected to genomic DNA extraction using a DTAB/CTAB DNA extraction kit (GeneReach Biotechnology Corp.). WSSV genomic DNA copies were quantified using IQ Real WSSV quantitative system (GeneReach Biotechnology Corp.), which is a commercial real-time PCR based on the TaqMan assay.

### Determination of the concentration of hemolymph lactate in shrimp pretreated with Rapamycin, Torin 1, LY294002 and MK2206 after WSSV infection

At 12 and 24 h post WSSV injection, 4–5 hemolymph samples (3 shrimp in each sample) were collected from groups of shrimp pretreated with LY294002, MK2206, Rapamycin, Torin 1 or PEG/PBS (control) without using anticoagulant. After being kept at 4°C for 12–16 hours, the samples were centrifuged at 13000×g for 15 min at 4°C, and the supernatants were transferred to new tubes. The concentration of glucose and lactate in the supernatants was then determined using enzymatic colorimetric test kits (Fortress Diagnostics Limited).

### Relative quantification of the expression of WSSV genes in shrimp pretreated with Rapamycin, Torin 1, LY294002, MK2206 and BKM120

After total hemocyte cDNA was prepared from all samples as described above, real-time PCR was performed with the specific primer sets IE1-qF/IE1-qR, DNApol-qF/DNApol-qR, VP28-qF/VP28-qR, ICP11-qF/ICP11-qR and EF1-α-qF/EF1-α-qR (Table S3) using the Bio-Rad detection system with Brilliant SYBR Green QPCR master mix (Applied Biosystems). Data values were calculated and presented as described above. Student's *t*-test was used to statistically analyze the Rapamycin and Torin 1 results. The LY294002 experiments used Tukey's multiple-comparison test (SPSS computer software) to evaluate statitiscally significant differences between experiemtnal groups.

### Quantification of WSSV genome copy number in shrimp pretreated with Rapamycin, Torin 1, LY294002 and MK2206

Genomic DNA was extracted from pleopod samples, and the number of WSSV genomic DNA copies was quantified by the IQ Real WSSV quantitative system (GeneReach Biotechnology Corp.) as described above. Data values were calculated, presented and statistically analyzed as described above.

### Protein extraction and western blot analysis

Shrimp gill tissues were lysed in 0.33× PBS with protein inhibitor and phosphatase inhibitor (Roche). Protein concentrations in each lysate were measured by Bio-Rad Protein Assay. Approximately 25 µg of protein lysate per sample were separated by 15% sodium dodecyl sulfate-polyacrylamide gel electrophoresis, transferred onto polyvinylidene fluoride (PDVF) membranes, blocked with 1–3% skim milk in Tris-buffered saline with 0.1% Tween 20 (TBST) for 1 hour at room temperature, and then incubated overnight in primary antibody in TBST at 4°C. Following three extensive washes with TBST, membranes were incubated with horseradish peroxidase (HRP)-conjugated secondary antibody (Santa Cruz) for 1 hour at room temperature. After three more washes with TBST, the signals were developed by ECL detection agents (Amersham) and detected using chemiluminescence (Image Quant LAS 4000 mini).

## Supporting Information

Figure S1 Hierarchical K-means Clustering of ∼800 hemocyte protein expression profiles obtained from a large-scale, high-throughput, label-free, quantitative LC-MS/MS analysis. (A), (B) At 12 hours and 24 hours post injection, the protein profiles of the PBS and WSSV groups formed two distinct clusters based on their log2 protein abundance, suggesting that the host cell protein pattern was markedly changed after WSSV infection at both time points. (C) Although there was no significant difference between the PBS groups at 12 and 24 hpi, the protein profiles of (D) the WSSV groups at 12 and 24 hpi formed two distinct clades, indicating that the host responses were different after WSSV infection at 12 and 24 hpi. Two of the samples, 12-WSSV#1 and 24-WSSV#2, were not assigned to the corresponding cluster, and we therefore excluded these two mis-assigned samples from our subsequent analysis.
(TIF)

Figure S2 Proteomic data suggests that the mTOR pathway is activated at the replication stage (12 hpi) of WSSV infection. (A) Changes in the levels of enzymes and proteins (ellipses) relative to PBS-injected controls are color-coded to represent up- (red) or down- (green) regulation. Yellow represents no change. Colorless ellipses indicate that no data was detected. (B) WSSV-induced phosphorylation of 4E-BP1 was still detected even after Rheb was knocked down by Rheb dsRNA. Each lane shows the results for a pooled sample (n = 3) of total protein extracted from gills and probes with antibodies against 4E-BP1-P^T37/46^, ICP11 and actin. (C) WSSV-induced phosphorylation of 4E-BP1 was suppressed by pretreatment with the inhibitor LY294002. Each lane shows the result for a pooled sample (n = 3) of total protein subjected to Western blotting with antibodies against 4E-BP1-P^T37/46^ and actin. (D) WSSV replication was significantly reduced by specifically suppressing using pretreatment with 0.625 µg/g shrimp of the selective pan-class I PI3K inhibitor BKM120 [45]. Data represent the mean ± SD of five pooled samples with each sample being taken from three different shrimp.
(TIF)

Figure S3 In Torin 1-pretreated shrimp, the Warburg effect was not seen either at 24 hpi in WSSV-infected shrimp or at 12∼24 hpi in PBS-injected shrimp. (A) Two hours after treatment with Torin 1, shrimp were injected with PBS or a WSSV inoculum. At 24 hpi, 6 pooled hemocytes samples (10 shrimp per pool) were collected from each group. Changes in the metabolomic levels of the WSSV-infected samples relative to the PBS controls are color-coded as described in Figure 1. Numerical data for 24 hpi is given in Table S2. (B) Effect of Torin 1 pretreatment at 12 and 24 h post PBS injection. The metabolic intermediates in Torin 1-pretreated shrimps injected with PBS were either down-regulated or remained unchanged. Changes in the metabolome for Torin 1-PBS versus PEG-PBS at 12 hpi and 24 hpi are shown in color-coded boxes as described in Figure 1, with numerical data given in Table S2.
(TIF)

Table S1 Global changes in the shrimp hemocyte proteome after WSSV infection.
(DOCX)

Table S2 Global changes in the shrimp hemocyte metabolome after WSSV infection.
(DOCX)

Table S3 PCR primers used in this study.
(DOCX)
