# Supplementary figures and images for: An Invertebrate Warburg Effect: A Shrimp Virus Achieves Successful Replication by Altering the Host Metabolome via the PI3K-Akt-mTOR Pathway
Source: PLoS Pathog. 2014 Jun 12;10(6):e1004196. doi: 10.1371/journal.ppat.1004196 (PMC4055789; doi:10.1371/journal.ppat.1004196)

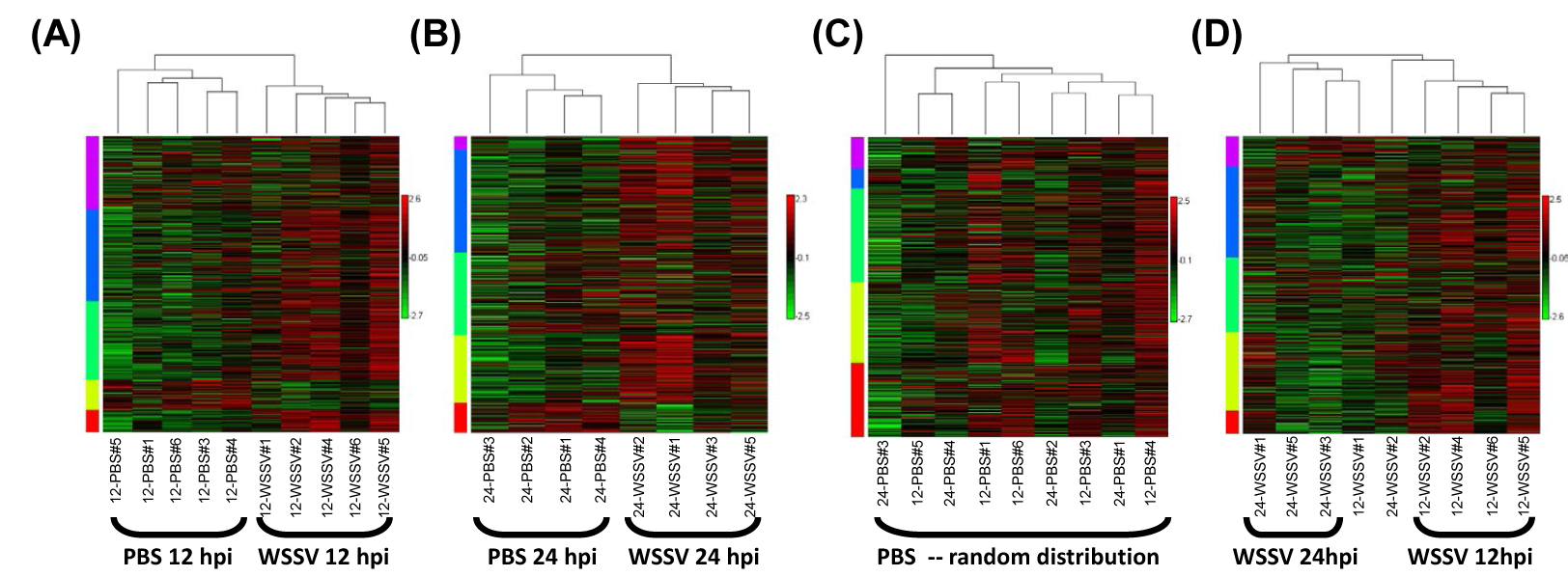

Supplement: Figure S1 — Hierarchical K-means Clustering of ∼800 hemocyte protein expression profiles obtained from a large-scale, high-throughput, label-free, quantitative LC-MS/MS analysis. (A), (B) At 12 hours and 24 hours post injection, the protein profiles of the PBS and WSSV groups formed two distinct clusters based on their log2 protein abundance, suggesting that the host cell protein pattern was markedly changed after WSSV infection at both time points. (C) Although there was no significant difference between the PBS groups at 12 and 24 hpi, the protein profiles of (D) the WSSV groups at 12 and 24 hpi formed two distinct clades, indicating that the host responses were different after WSSV infection at 12 and 24 hpi. Two of the samples, 12-WSSV#1 and 24-WSSV#2, were not assigned to the corresponding cluster, and we therefore excluded these two mis-assigned samples from our subsequent analysis. (TIF) [file ppat.1004196.s001.tif]

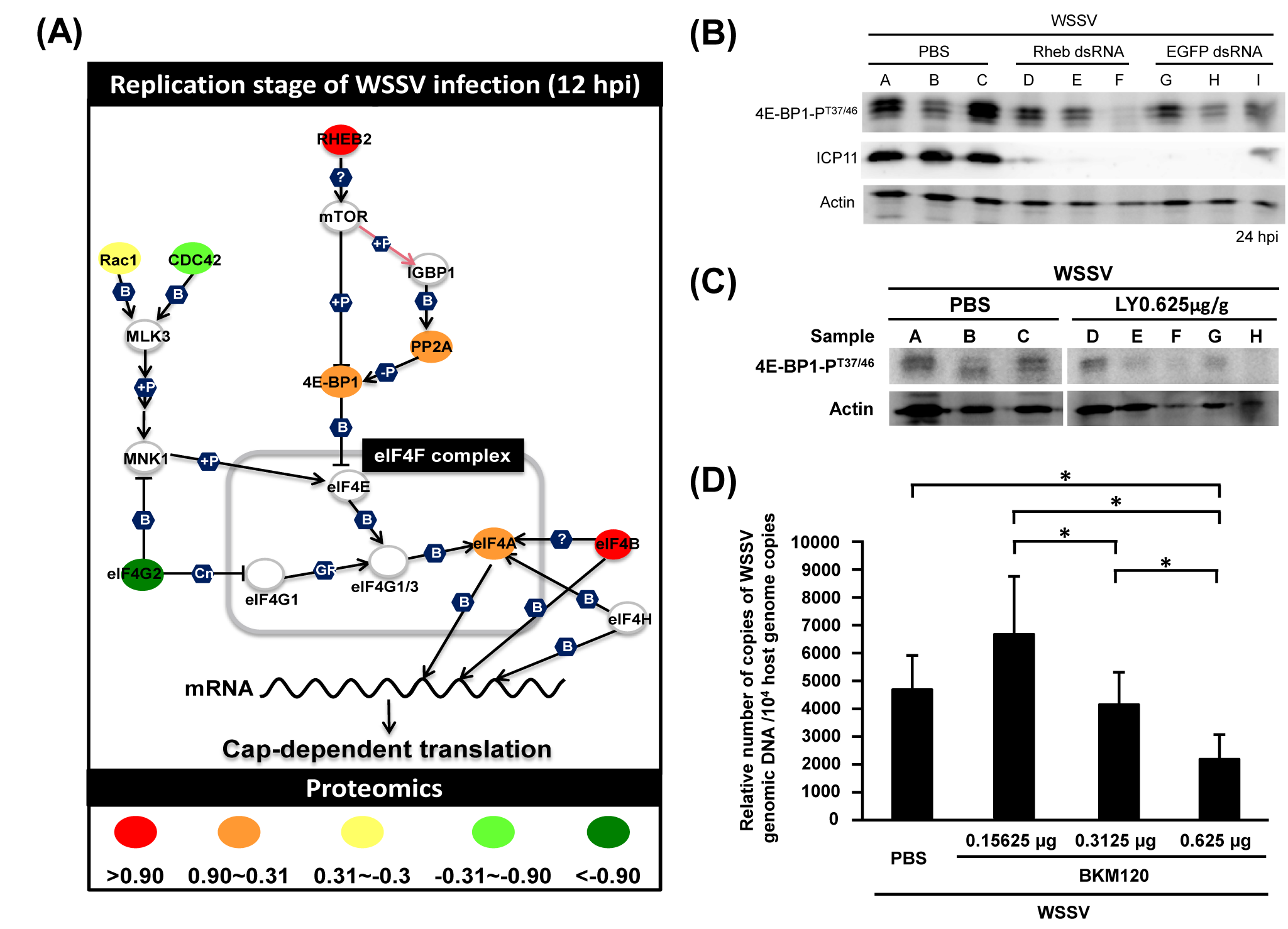

Supplement: Figure S2 — Proteomic data suggests that the mTOR pathway is activated at the replication stage (12 hpi) of WSSV infection. (A) Changes in the levels of enzymes and proteins (ellipses) relative to PBS-injected controls are color-coded to represent up- (red) or down- (green) regulation. Yellow represents no change. Colorless ellipses indicate that no data was detected. (B) WSSV-induced phosphorylation of 4E-BP1 was still detected even after Rheb was knocked down by Rheb dsRNA. Each lane shows the results for a pooled sample (n = 3) of total protein extracted from gills and probes with antibodies against 4E-BP1-PT37/46, ICP11 and actin. (C) WSSV-induced phosphorylation of 4E-BP1 was suppressed by pretreatment with the inhibitor LY294002. Each lane shows the result for a pooled sample (n = 3) of total protein subjected to Western blotting with antibodies against 4E-BP1-PT37/46 and actin. (D) WSSV replication was significantly reduced by specifically suppressing using pretreatment with 0.625 µg/g shrimp of the selective pan-class I PI3K inhibitor BKM120 [45]. Data represent the mean ± SD of five pooled samples with each sample being taken from three different shrimp. (TIF) [file ppat.1004196.s002.tif]

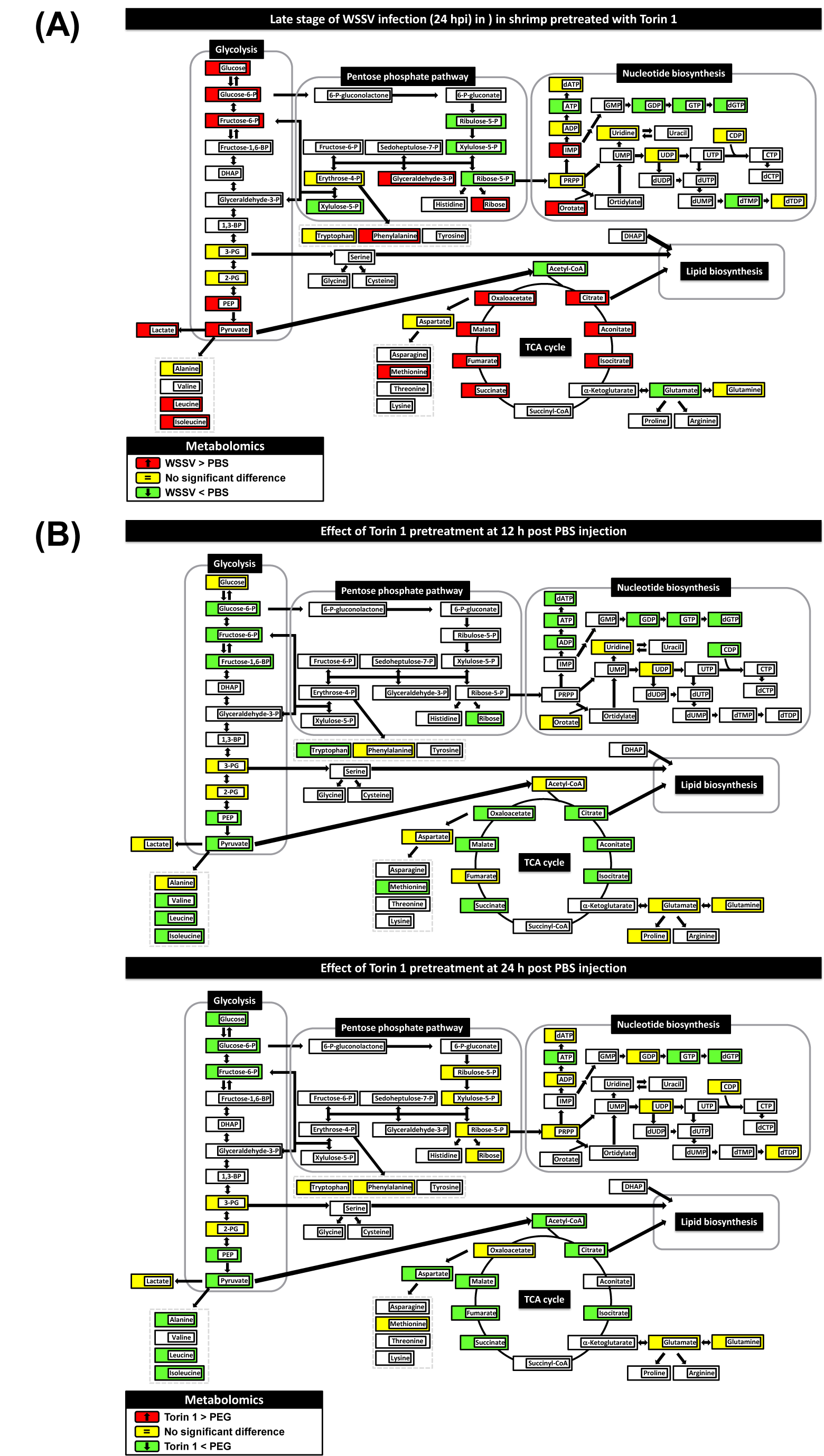

Supplement: Figure S3 — In Torin 1-pretreated shrimp, the Warburg effect was not seen either at 24 hpi in WSSV-infected shrimp or at 12∼24 hpi in PBS-injected shrimp. (A) Two hours after treatment with Torin 1, shrimp were injected with PBS or a WSSV inoculum. At 24 hpi, 6 pooled hemocytes samples (10 shrimp per pool) were collected from each group. Changes in the metabolomic levels of the WSSV-infected samples relative to the PBS controls are color-coded as described in Figure 1. Numerical data for 24 hpi is given in Table S2. (B) Effect of Torin 1 pretreatment at 12 and 24 h post PBS injection. The metabolic intermediates in Torin 1-pretreated shrimps injected with PBS were either down-regulated or remained unchanged. Changes in the metabolome for Torin 1-PBS versus PEG-PBS at 12 hpi and 24 hpi are shown in color-coded boxes as described in Figure 1, with numerical data given in Table S2. (TIF) [file ppat.1004196.s003.tif]
