# Supplementary material for: An Invertebrate Warburg Effect: A Shrimp Virus Achieves Successful Replication by Altering the Host Metabolome via the PI3K-Akt-mTOR Pathway
Source: PLoS Pathog. 2014 Jun 12;10(6):e1004196. doi: 10.1371/journal.ppat.1004196 (PMC4055789; doi:10.1371/journal.ppat.1004196)
Supplement: Table S1 — Global changes in the shrimp hemocyte proteome after WSSV infection. (DOCX) [file ppat.1004196.s004.docx]

**Table S1. Global changes in the shrimp hemocyte proteome after WSSV infection**

| **Pathway** | **Protein symbol** | **Description** | **log_2_ (WSSV vs PBS)** | | | |
| --- | --- | --- | --- | --- | --- | --- |
|  |  |  | **12 hpi** | | **24hpi** | |
|  |  |  | **Excl. #1** | **Incl. #1*** | **Excl. #2** | **Incl. #2*** |
| Glycolysis | HK | Hexokinase | 1.63 | 1.72 | 1.1 | 0.8 |
|  | GPI | Glucose-6-phosphate isomerase | 0.34 | 0.27 | 0.2 | -0.4 |
|  | F16P | Fructose-1,6-bisphosphatase | 0.51 | 0.43 | 0.24 | 0.26 |
|  | ALDOA | Fructose-bisphosphate aldolase | 1.15 | 1.02 | 0.43 | 0.45 |
|  | TPI | Triosephosphate isomerase | 0.79 | 0.67 | 0.39 | 0.38 |
|  | G3P2 | Glyceraldehyde-3-phosphate dehydrogenase | 0.83 | 0.71 | 0.54 | 0.62 |
|  | PGK | Phosphoglycerate kinase | 0.12 | 0.03 | 0.2 | 0.18 |
|  | PGAM | Phosphoglycerate mutase | 0.6 | 0.47 | 0.24 | 0.2 |
|  | ENO | Enolase | 0.94 | 0.83 | 1.08 | 0.79 |
|  | PK | Pyruvate kinase | 1.04 | 0.88 | 0.52 | 0.54 |
| Pentose phosphate pathway | RPE | Ribulose-phosphate 3-epimerase | 0.83 | 0.66 | 1 | 1.08 |
|  | TALDO | Transaldolase | 0.56 | 0.51 | 0.49 | 0.42 |
|  | TKT | Transketolase | 0.73 | 0.71 | 0.55 | 0.46 |
|  | PGMU | Phosphoglucomutase | 0.73 | 0.69 | 0.73 | 0.66 |
| Glutamine metabolism | GLNA | Glutamine synthetase | -2.85 | -3.10 | -2.25 | -2.6 |
|  | DHE3 | Glutamate dehydrogenase | 0.73 | 0.70 | 0.9 | 0.8 |
| TCA cycle | MDH2 | Malate dehydrogenase, mitochondria | 0.54 | 0.57 | 0.38 | 0.27 |
|  | FUMH | Fumarate hydratase,mitochondria | 0.61 | 0.63 | -1.04 | 0.62 |
|  | SDH | Succinate dehydrogenase | -0.89 | -0.52 | -1.15 | -0.97 |
|  | CISY | Citrate synthase | 0.86 | 0.87 | 0.79 | 0.79 |
|  | DLDH | Dihydrolipoyl dehydrogenase | 0.45 | 0.78 | 0.38 | 0.33 |
|  | IDH2 | Isocitrate dehydrogenase | 0.74 | 0.50 | 1.44 | 1.37 |
|  | IDH3 | Isocitrate dehydrogenase 3 complex | 1.16 | 1.10 | 0.86 | 0.8 |
|  | SCS-A^#^ | Succinyl-CoA ligase subunit A complex | -2.57 | -2.51 | -0.53 | -2.79 |
|  | SCS-G^#^ | Succinyl-CoA ligase subunit G complex | -2.57 | -2.51 | 0.13 | -2.79 |
|  | SUCLG1 | Succinyl-CoA ligase subunit alpha (GDP-forming) | -2.57 | -2.51 | 0.46 | -2.79 |
|  | SUCLG2 | Succinyl-CoA ligase subunit beta (GDP-forming) | -0.07 | 0.12 | 0.13 | 0.03 |
|  | SUCB1 | Succinyl-CoA ligase subunit beta (ADP-forming) | -0.75 | -0.65 | -0.53 | -0.59 |
| Translation-Regulation of EIF4F activity | Rac1 | Ras-related C3 botulinum toxin substrate 1 | 0.04 | -0.10 | 0.17 | 0.17 |
|  | CDC42 | Cell division cycle 42 | -0.44 | -0.55 | -0.23 | -0.23 |
|  | RHEB2 | GTP-binding protein Rheb | 3.18 | 2.92 | -0.35 | -0.35 |
|  | PP2A | Protein phosphatase 2A catalytic protein group | 0.64 | 0.22 | 0.49 | 0.49 |
|  | 4E-BP1 | Eukaryotic translational initiation factor 4E-binding protein 1 | 0.64 | 0.48 | 0.79 | 0.79 |
|  | eIF4G2 | Eukaryotic translation initiation factor 4 gamma 2 | -1.38 | -1.29 | -1.38 | -1.38 |
|  | eIF4A | Eukaryotic translation initiation factor 4A | 0.89 | 0.86 | 0.68 | 0.68 |
|  | eIF4B | Eukaryotic translation initiation factor 4B | 0.97 | 0.94 | 0.75 | 0.82 |

* In the clustering analysis (Fig. S1) the WSSV 12 hpi sample #1 and the WSSV 24 hpi sample #2 were mis-assigned and therefore excluded from subsequent analysis. The two “Incl” columns show the data values that result when these two anomalous samples are included.

^#^ SCS-A and SCS-G are complexes formed from SUCLG1 and SUCB1, and from SUCLG1 and SUCLG2, respectively. These two complexes were not measured directly. Instead they were assumed ti be limited by the availability of their constituents, and they were therefore assigned the same values as their lowest constituent protein.
