# Supplementary material for: An Invertebrate Warburg Effect: A Shrimp Virus Achieves Successful Replication by Altering the Host Metabolome via the PI3K-Akt-mTOR Pathway
Source: PLoS Pathog. 2014 Jun 12;10(6):e1004196. doi: 10.1371/journal.ppat.1004196 (PMC4055789; doi:10.1371/journal.ppat.1004196)
Supplement: Table S2 — Global changes in the shrimp hemocyte metabolome after WSSV infection. (DOCX) [file ppat.1004196.s005.docx]

Red and green indicate significant up- and down-regulation, respectively.

**Table S2. Global changes in the shrimp hemocyte metabolome after WSSV infection**

| Super-  pathway | Sub-  pathway | Biochemical name | PEG pretreatment  (fold change [WSSV / PBS]) | | Torin1 pretreatment  ( fold change [WSSV / PBS]) | | PBS injection  ( fold change [ Torin 1 / PEG ]) | |
| --- | --- | --- | --- | --- | --- | --- | --- | --- |
|  |  |  | 12 hpi | 24 hpi | 12 hpi | 24 hpi | 12 hpi | 24 hpi |
| Amino Acid | Alanine and aspartate metabolism | alanine | 1.619014628 | 0.986677817 | 1.811118962 | 1.231283925 | 0.73202251 | 0.657676605 |
|  |  | aspartate | 2.010206129 | 1.116002516 | 2.036355034 | 1.079392165 | 0.80339138 | 0.717750637 |
|  |  | asparagine | 1.57623383 | ∞ | ∞ | n/d | n/d | n/d |
|  | Glutamate metabolism | glutamate | 3.477321922 | 0.623259821 | 3.192441299 | 0 | 0.714375771 | 0.242027337 |
|  |  | glutamine | 1.014457219 | 0.986294168 | 0.660845378 | 0.825948226 | 1.01210606 | 0.801223291 |
|  | Phenylalanine & tyrosine metabolism | phenylalanine | 2.035372649 | 1.804400115 | 3.975296618 | 1.891547235 | 0.544517462 | 1.023122592 |
|  | Tryptophan metabolism | tryptophan | 3.790966712 | 1.52192301 | 5.680191779 | 1.037891594 | 0.44971009 | 0.742252355 |
|  | Valine, leucine and isoleucine metabolism | isoleucine | 0.6802267 | 0.45983868 | 1.36889638 | 1.4981927 | 0.643735593 | 0.28808886 |
|  |  | leucine | 0.6802267 | 0.45983868 | 1.36889638 | 1.4981927 | 0.643735593 | 0.28808886 |
|  |  | valine | 0.663814561 | 1.18736301 | 2.140211067 | n/d | 0.622080481 | n/d |
|  | Cysteine, methionine, SAM, taurine metabolism | cysteine | n/d | n/d | 0 | n/d | n/d | n/d |
|  |  | methionine | 1.349376134 | 1.909035252 | 3.027039095 | 3.939140593 | 0.331870388 | 0.623273355 |
|  | Proline metabolism | proline | 2.270670918 | n/d | 2.619280124 | n/d | 0.979139625 | n/d |
| Carbohydrate | Sucrose metabolism | sucrose | ∞ | 1.655595081 | 3.128611782 | 3.695657498 | n/d | 0.914654757 |
|  | Glycolysis, gluconeogenesis, pyruvate metabolism | glucose-6-phosphate (G6P) | 2.819054207 | 0.965331992 | 3.427805545 | 3.672442793 | 0.598051892 | 0.300924876 |
|  |  | glucose 1-phosphate | 2.819054207 | 0.965331992 | 3.427805545 | 3.672442793 | 0.598051892 | 0.300924876 |
|  |  | glucose | 1.442878268 | 0.859593284 | 0.817415403 | 2.203495491 | 1.209111475 | 0.468075768 |
|  |  | fructose-6-phosphate | 2.819054207 | 0.965331992 | 3.427805545 | 3.672442793 | 0.598051892 | 0.300924876 |
|  |  | fructose 1,6-diphosphate, glucose 1,6-diphosphate | 0.909762144 | n/d | 1.067015603 | n/d | n/d | n/d |
|  |  | 2-phosphoglycerate | 1.42047441 | 0.822782734 | 0.97676898 | 0.905328462 | 0.437477671 | n/d |
|  |  | 3-phosphoglycerate | 1.42047441 | 0.822782734 | 0.97676898 | 0.905328462 | 1.189292796 | 0.771669253 |
|  |  | dihydroxyacetone phosphate (DHAP) | ∞ | n/d | ∞ | n/d | 1.189292796 | 0.771669253 |
|  |  | D-Glyceraldehyde 3-phosphate | ∞ | n/d | ∞ | n/d | n/d | n/d |
|  |  | phosphoenolpyruvate (PEP) | 1.134255455 | 0.419078872 | 1.399926365 | 1.355935101 | 0.709003552 | 0.831647159 |
|  |  | pyruvate | 1.71219204 | 2.028269477 | 2.262346 | 3.314350751 | 0.489611857 | 0.665564188 |
|  |  | lactate(Hcy) | 2.035372649 | 1.804400115 | 3.975296618 | 1.891547235 | 0.544517462 | 1.023122592 |
|  | Nucleotide sugars, pentose metabolism | Phosphoribosyl pyrophosphate (PRPP) | 0 | 0 | ∞ | 1.165508185 | n/d | n/d |
|  |  | 5-Phosphoribosylamine (PRA) | 2.193808082 | n/d | ∞ | 0.739337952 | n/d | n/d |
|  |  | ribose | 2.433553339 | 4.154004983 | 2.764934704 | 3.061604128 | 0.650516782 | 1.136111856 |
|  |  | ribose 5-phosphate/ribose 1-phosphate | ∞ | 0 | n/d | 0 | n/d | 0.943362036 |
|  |  | ribulose 5-phosphate, xylulose 5-phosphate | ∞ | 0 | n/d | 0 | n/d | 0.943362036 |
|  |  | D-Erythrose 4-phosphate | ∞ | n/d | 1.447347356 | 0.602004746 | n/d | n/d |
| Energy | Krebs cycle | Acetyl-CoA | 0.522866393 | 1.00672927 | 0.698378965 | 0 | 0.525412817 | 0.348726082 |
|  |  | citrate | 1.479800249 | 0.998134166 | 4.268164643 | 4.231423693 | 0.362292224 | 0.296079698 |
|  |  | cis-Aconitic acid | 3.44011925 | 0.690781076 | 1.872323176 | ∞ | 0.505871668 | n/d |
|  |  | succinate | 1.169447921 | 1.832354848 | 1.979727732 | 2.645295988 | 0.457512738 | 0.646609413 |
|  |  | fumarate | 2.291337009 | 2.434433292 | 2.283651533 | 5.063090551 | 0.726716441 | 0.511214776 |
|  |  | malate | 1.745067279 | 1.958028802 | 2.513891972 | 3.810297459 | 0.465514519 | 0.556742311 |
|  |  | Oxalacetic acid | 1.815054247 | 2.334786905 | 5.504704119 | 2.493763633 | 0.256205789 | 0.795939045 |
| Nucleotide | Purine metabolism, (hypo)xanthine / inosine containing | xanthine | 1.402288547 | 1.765421527 | 1.36402105 | 4.051816759 | n/d | n/d |
|  |  | hypoxanthine | 1.40522855 | ∞ | 1.395788193 | 0 | n/d | n/d |
|  |  | inosine | 0 | 1.432516749 | 0 | ∞ | n/d | n/d |
|  | Purine metabolism, adenine containing | adenine | 2.312655044 | 0.810454449 | 2.665452874 | 1.165923982 | 0.862361528 | 0.296709683 |
|  |  | adenosine 5'-diphosphate (ADP) | 1.389496963 | 1.018422261 | 1.109561601 | 0.944393404 | 0.860551529 | 0.932064954 |
|  |  | adenosine 5'-triphosphate (ATP) | 1.551115779 | 0.622291912 | 1.350168019 | 0.29703549 | 0.832496188 | 0.674584651 |
|  |  | Deoxyadenosine triphosphate (dATP) | 1.19396953 | 1.037376722 | 1.539211253 | 0.757056374 | 0.556901166 | 1.580575681 |
|  | Purine metabolism, guanine containing | guanine | 1.498005859 | n/d | n/d | n/d | n/d | n/d |
|  |  | Guanosine diphosphate (GDP) | 1.423614799 | 1.068708858 | 1.194308515 | 0.787104233 | 0.839428334 | 1.031033823 |
|  |  | Guanosine triphosphate (GTP) | 1.527764928 | 0.540465887 | 1.334896017 | 0.176032372 | 0.743387023 | 0.64847617 |
|  |  | Deoxyguanosine triphosphate(dGTP) | 1.551115779 | 0.622291912 | 1.350168019 | 0.29703549 | 0.832496188 | 0.674584651 |
|  | Pyrimidine metabolism, cytidine containing | Cytidine | n/d | 0 | n/d | 0 | n/d | 0.708466213 |
|  |  | cytidine monophosphate (CDP) | 1.167598464 | 1.022603466 | 1.148510767 | 1.041279467 | 0.76240316 | 0.8726397 |
|  | Pyrimidine metabolism, uracil containing | uracil | ∞ | n/d | n/d | n/d | n/d | n/d |
|  |  | uridine | 3.35884421 | ∞ | 1.138472934 | 1.464970744 | 1.14499668 | n/d |
|  |  | Uridine 5'-diphosphate (UDP) | 1.508654581 | 1.053474393 | 1.30794915 | 0.882539941 | 0.861873893 | 0.92733014 |
|  |  | Uric acid | 2.486330466 | 0.930910923 | 1.024498743 | 1.287331855 | 2.460674751 | 0.782881397 |
|  | Thymidine metabolism | Thymidine | ∞ | n/d | n/d | n/d | n/d | n/d |
|  |  | Thymidine diphosphate (dTDP) | ∞ | 1.494418782 | 1.188187901 | 1.712461406 | n/d | 0.697889417 |
|  | Pyrimidine metabolism, thymine containing | Dihydrothymine | 0.71300976 | n/d | 1.471226408 | n/d | 0.394267505 | n/d |
|  |  | Ureidoisobutyric acid | 1.014457219 | 0.986294168 | 0.660845378 | 0.825948226 | 1.01210606 | 0.801223291 |

Yellow indicates no significant change.

n/d: not detected

0: not detected in WSSV-infected cells

∞: only detected in WSSV-infected cells
