# Supplementary material for: An Invertebrate Warburg Effect: A Shrimp Virus Achieves Successful Replication by Altering the Host Metabolome via the PI3K-Akt-mTOR Pathway
Source: PLoS Pathog. 2014 Jun 12;10(6):e1004196. doi: 10.1371/journal.ppat.1004196 (PMC4055789; doi:10.1371/journal.ppat.1004196)
Supplement: Table S3 — PCR primers used in this study. (DOCX) [file ppat.1004196.s006.docx]

**Table S3. PCR primers used in this study**

| Gene | Primer name | Primer sequence (5'-3') | Usage |
| --- | --- | --- | --- |
| *LvRheb* |  |  |  |
|  | LvRheb-F531 | 5'-CCATCGAGAACACCTTCA-3' | dsRNA synthesis |
|  | LvRheb-R882 | 5'-GCCTCATGTTGTTTGGCA-3' | dsRNA synthesis |
|  | LvRheb-dsT7F531 | 5'-TAATACGACTCACTATAGGGAGACCATCGAGAACACCTTCA-3' | dsRNA synthesis |
|  | LvRheb-dsT7R882 | 5'-TAATACGACTCACTATAGGGAGAGCCTCATGTTGTTTGGCA-3' | dsRNA synthesis |
|  | LvRheb-qF | 5'-TGCCCCAACCATCGAGAA-3' | Real-time PCR |
|  | LvRheb-qR | 5'-GCCATACTCCTGCCCTCTCA-3' | Real-time PCR |
| EGFP |  |  |  |
|  | EGFP dsF | 5'-GTTCAGCGTGTCCGGCGAG-3' | dsRNA synthesis |
|  | EGFP dsR | 5'-GTTCTTCTGCTTGTCGGCC-3' | dsRNA synthesis |
|  | EGFP dsT7F | 5'-TAATACGACTCACTATAGGGAGAGTTCAGCGTGTCCGGCGAG-3' | dsRNA synthesis |
|  | EGFP dsT7R | 5'-TAATACGACTCACTATAGGGAGAGTTCTTCTGCTTGTCGGCC-3' | dsRNA synthesis |
| *EF1-α* |  |  |  |
|  | EF1-α-qF | 5'-ACGTGTCCGTGAAGGATCTGAA-3' | Real-time PCR |
|  | EF1-α-qR | 5'-TCCTTGGCAGGGTCGTTCTT-3' | Real-time PCR |
| WSSV IE1 |  |  |  |
|  | IE1-qF | 5'-CCAGGCCCAGTGTCATACG-3' | Real-time PCR |
|  | IE1-qR | 5'-AGAAATCTCATCACATGTCAAATCAGA-3' | Real-time PCR |
| WSSV DNA pol |  |  |  |
|  | DNA pol -qF | 5'-CGTTTGATTATGCGGGTGCT-3' | Real-time PCR |
|  | DNA pol -qR | 5'-TCTCGTGGTGGCACCATCT-3' | Real-time PCR |
| WSSV VP28 |  |  |  |
|  | VP28-qF | 5'-AGTTGGCACCTTTGTGTGTGGTA-3' | Real-time PCR |
|  | VP28-qR | 5'-TTTCCACCGGCGGTAGCT-3' | Real-time PCR |
| WSSV ICP11 |  |  |  |
|  | ICP11 -qF | 5'-TTGAGGCAGTCAGGAAGAGTGA-3' | Real-time PCR |
|  | ICP11 -qR | 5'- GGCACACCATGTAAACACGGT-3' | Real-time PCR |
| Others |  |  |  |
|  | Anchor dTv | 5'-GACCACGCGTATCGATGTCGACTTTTTTTTTTTTTTTTV-3' | cDNA synthesis |

The added T7 promoter sequence is underlined.
